# Exploring Novel Antimalarial Compounds Targeting *Plasmodium falciparum* Enoyl-ACP Reductase: Computational and Experimental Insights

**DOI:** 10.1021/acsomega.3c09893

**Published:** 2024-05-13

**Authors:** George A R Oliveira, Bruno G D V Morales, Rosa M O Sousa, Soraya S Pereira, Deborah Antunes, Ernesto R. Caffarena, Fernando B. Zanchi

**Affiliations:** † Laboratório de Bioinformática e Química Medicinal, Fundação Oswaldo Cruz, CEP: 76812-245 Porto Velho-RO, Brazil; ‡ Programa de Pós-Graduação em Biologia Experimental, Fundação Universidade Federal de Rondônia (UNIR), CEP: 76801-974 Porto Velho-RO, Brazil; § Instituto Nacional de Epidemiologia na Amazônia Ocidental—EPIAMO, CEP: 76812-245 Porto Velho-RO, Brazil; ∥ Laboratório de Engenharia de Anticorpos, Fundação Oswaldo Cruz de Rondônia, CEP: 76812-245 Porto Velho-RO, Brazil; ⊥ Programa de Pós-graduação Stricto sensu em Biologia Computacional e Sistemas do Instituto Oswaldo Cruz, CEP: 21040-360 Rio de Janeiro-RJ, Brazil; # Laboratório de Genômica Aplicada e Bioinovações, Instituto Oswaldo Cruz, Fundação Oswaldo Cruz (FIOCRUZ), CEP: 21040-900 Rio de Janeiro-RJ, Brazil; ∇ Programa de Computação Científica—PROCC, Fundação Oswaldo Cruz, CEP: 21040-900 Rio de Janeiro-RJ, Brazil

## Abstract

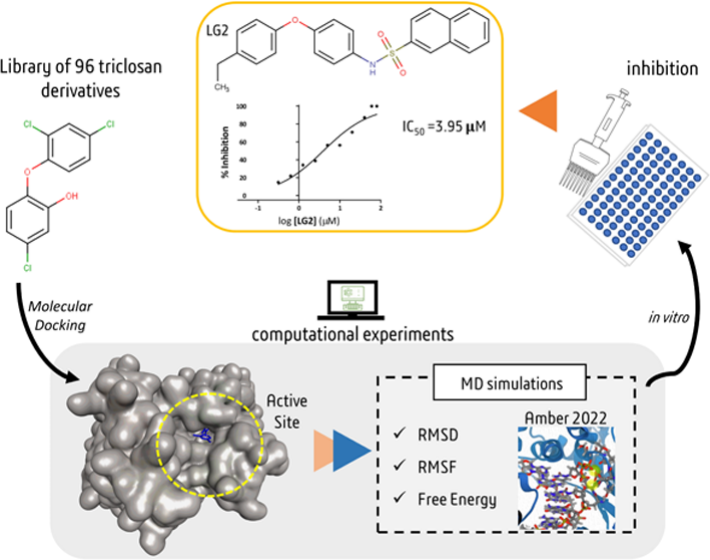

Malaria, caused by Plasmodium protozoa with *Plasmodium falciparum* as the most virulent species, continues to pose significant health challenges. Despite the availability of effective antimalarial drugs, the emergence of resistance has heightened the urgency for developing novel therapeutic compounds. In this study, we investigated the enoyl-ACP reductase enzyme of *P. falciparum* (PfENR) as a promising target for antimalarial drug discovery. Through a comprehensive analysis, we conducted a comparative evaluation of two lead compounds, LD1 (CID: 44405336, lead compounds 1) and LD2 (CID: 72703246, lead compounds 2), obtained from the PubChem/NCBI ligand database, to serve as reference molecules in the identification of potential derivatives using virtual screening assays. Among the newly identified candidates, Ligand 1 (LG1) and Ligand 2 (LG2) exhibited intriguing characteristics and underwent further investigation through docking and molecular dynamics simulations. Ligand 1 (LG1) demonstrated interactions similar to LD1, including hydrogen bonding with Asp218, while Ligand 2 (LG2) displayed superior binding energy comparable to LD1 and LD2, despite lacking hydrogen bonding interactions observed in the control compounds triclosan and its derivative 7-(4-chloro-2-hydroxyphenoxy)-4-methyl-2H-chromen-2-one (CHJ). Following computational validation using the MM/GBSA method to estimate binding free energy, commercially acquired LG1 and LG2 ligands were subjected to in vitro testing. Inhibition assays were performed to evaluate their potential as PfENR inhibitors alongside triclosan as a control compound. LG1 exhibited no inhibitory effects, while LG2 demonstrated inhibitory effects like triclosan. In conclusion, this study contributes valuable insights into developing novel antimalarial drugs by identifying LG2 as a potential ligand and employing a comprehensive approach integrating computational and experimental methodologies.

## 1 Introduction

Malaria, caused by various *Plasmodium* species, including *Plasmodium vivax*, *Plasmodium ovale*, *Plasmodium malariae*, and *Plasmodium falciparum*, remains a significant global health concern.^1^ In 2020, Africa witnessed a staggering number of malaria cases, with approximately 241 million reported, leading to 627 000 deaths, according to the World Health Organization (WHO). Similarly, countries such as Bolivia and Venezuela in the Americas experienced a substantial increase in malaria cases, reaching over 467 000 cases in 2019. However, there was a notable decline in cases in 2020, potentially influenced by the COVID-19 pandemic and other factors such as reduced occupational exposure risks.^2^

Despite the availability of efficacious antimalarial medications and preventive measures such as insecticide-treated bed nets (ITNs), malaria remains a daunting threat, particularly in impoverished regions.^3^ Among the *Plasmodium* species, *P. falciparum* stands out as the most virulent, causing severe manifestations of the disease and posing a significant risk of mortality if left untreated.^4^

In Brazil, the Ministry of Health has recommended artemisinin-based combination therapies (ACTs) as the primary treatment for *falciparum* malaria, with combinations like artesunate/mefloquine and artemether/lumefantrine demonstrating significant efficacy against *P. falciparum* infections.^5^ However, the widespread adoption of these treatments is influenced by factors such as pharmaceutical availability and individual patient-specific requirements, limitations, or financial constraints.^6^

Nevertheless, the emergence of drug resistance has severely compromised the effectiveness of current malaria therapies, including widely used drugs, such as chloroquine and artemisinin, leading to increased morbidity and mortality rates. Overcoming drug resistance is an urgent priority, emphasizing the critical need to discover and develop new therapeutic agents against *P. falciparum* infections.^2,7^

Extensive research efforts have focused on exploring inhibitors that selectively target various enzymes and receptors within the malaria parasite, aiming to identify crucial molecular targets for potential therapeutic compounds. A comprehensive understanding of the biochemistry underlying these targets enables the identification of molecules capable of efficiently inhibiting their activity, disrupting essential metabolic processes, and ultimately leading to parasite eradication. In the context of combatting malaria and related diseases, enzymes participating in the fatty acid biosynthesis pathway, particularly the enoyl-ACP reductase (ENR) enzyme, have emerged as a compelling candidate for drug targeting.^8,9^ The Plasmodium’s fatty acid synthesis (FAS) system, notably the FAS II system, offers an opportunity to selectively address enzymes involved in fatty acid biosynthesis as potential drug targets against malaria. ENR, a pivotal enzyme within this pathway, governs the elongation cycle of fatty acids and catalyzes the principal reaction in fatty acid biosynthesis.^10,11^

Considering the established evidence that enoyl-ACP reductase (ENR) plays a pivotal role in determining the rate of type II fatty acid synthesis (FAS type II), it becomes apparent that ENR holds significant importance in regulating fatty acid catalysis and bacterial cell wall biosynthesis. Inhibiting fatty acid synthesis through ENR inhibition is anticipated to have the potential to impair the survival capabilities of *P. falciparum*, offering a promising avenue for therapeutic intervention.^12^

In drug discovery, innovative strategies combine computational and experimental methodologies. Structure-based drug design (SBDD) and ligand-based drug design (LBDD) techniques play essential roles in the drug design process, leveraging advanced techniques such as X-ray crystallography and nuclear magnetic resonance (NMR) spectroscopy to generate molecular structure databases, enabling virtual screening, docking studies, and pharmacokinetic investigations.^13^

Among the compounds under investigation as inhibitors of *Plasmodium* molecular targets, triclosan and its derivatives have received significant attention due to their inhibitory action against *P. falciparum* 2-trans-enoyl-ACP reductase (PfENR). Triclosan, known for its bactericidal properties, has been widely employed in consumer products and demonstrated inhibitory effects on ENR in both *Escherichia coli* and PfENR.^14−16^ Triclosan (TCL) is a widely used antimicrobial found in numerous personal care products, exposing around 75% of the US population. Following a risk assessment in September 2016, the FDA banned its use in soaps.^17^

Studies have shown that TCL can induce effects shortly after chemical exposure. It can undergo both biotic and abiotic transformations, leading to the formation of various byproducts. Some of these byproducts remain inadequately investigated, and certain ones have been identified as more toxic than the original TCL. Additionally, TCL has been linked to endocrine-disrupting effects and has shown high toxicity levels in mouse studies.^18,19^ Therefore, exploring triclosan-derived compounds with increased potency and favorable pharmacological properties as PfENR inhibitors is a rational approach.^20^

In this study, we utilized in silico prospecting techniques, recombinant protein expression, and purification to perform molecular interaction assays to identify potential inhibitors for PfENR. The research presented herein offers insights into alternative treatment options for malaria, contributing to ongoing efforts in combating this global health burden.

## 2 Materials and Methods

**Figure 1:**
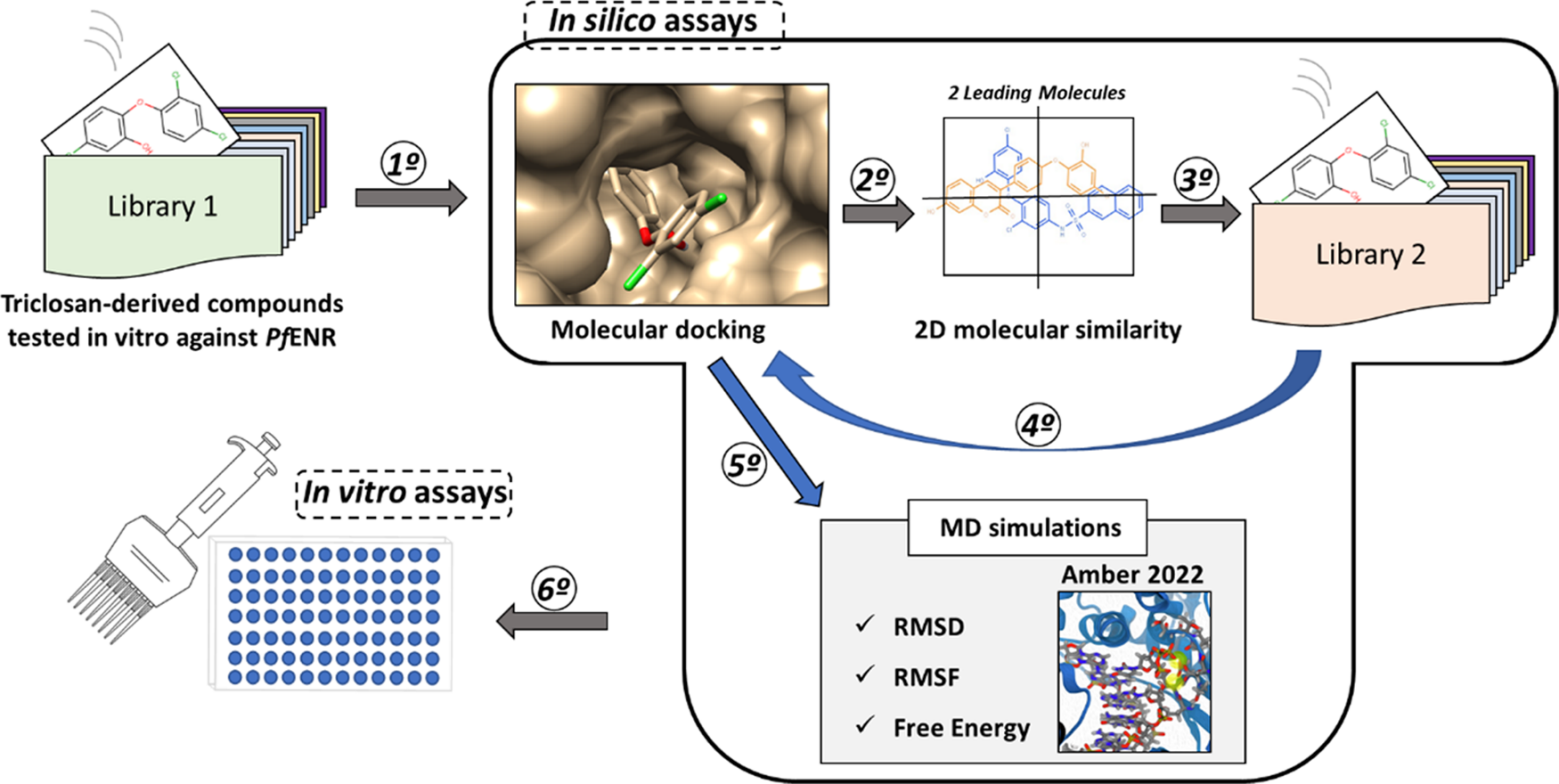
Workflow for the identification of potential antimalarial drug candidates. Triclosan-derived compounds from Library 1 were subjected to molecular docking against PfENR. The two ligands with the most favorable binding energies were selected as lead compounds for virtual screening (VS) assays to identify novel compounds. The top-scoring candidates from the VS were further evaluated through molecular dynamics simulations and subsequently tested in vitro for inhibitory activity.

The initial stage involved an extensive literature search to identify triclosan derivatives that had previously been assessed in vitro against PfENR, resulting in the selection of 96 molecules. These compounds’ three-dimensional (3D) structures were obtained from PubChem, thus forming the initial library known as Library 1. The outcomes of redocking proved to be highly satisfactory (RMSD < 2 A) when evaluating the performance of both docking software in replicating the ligand CHJ’s binding within the crystallographic structure (PDB 4IGE). Therefore, molecular docking utilizing Library 1 was performed on the 3D structure of PfENR to identify lead compounds, employing the AutoDock Vina program. The two compounds displaying the most favorable binding energies were selected as lead candidates. In the subsequent step, a search was conducted in PubChem to identify structurally similar molecules, forming the basis of the second library (Library 2). This library, consisting of 335 newly obtained molecules, underwent virtual screening via molecular docking using the Autodock Vina and DockThor programs (Figure 1). These molecules’ top two candidates were chosen for further analysis through molecular dynamics simulations. Finally, in vitro inhibitory tests were performed to evaluate their inhibitory activity.

### 2.1 PfENR Structure Intrinsically Disordered Regions, Secondary Structure Predictions, and Comparative Model Construction

The amino acid sequence of PfENR was retrieved from the National Center for Biotechnology Information (NCBI) database under accession number XP 966137. The three-dimensional structure was obtained through a similarity search against sequences within the Protein Data Bank (PDB) using the BLAST tool, with selection based on the criterion of the highest coverage and resolution.^21^

The deposited PfENR structures in the Protein Data Bank (PDB) exhibit an unresolved region of approximately 40 amino acids. The XP 966137 sequence was submitted to the DISOPRED3,^22^ PrDOS,^23^ and Predict Protein^24^ servers to investigate if this region corresponds to an intrinsically disordered region (IDR). Secondary structure predictions were obtained from the PSIPRED,^25^ Predict Protein,^24^ and JPred4^26^ servers. A small portion of amino acids (349–361) from XP 966137 were aligned against the sequence of the PDB file (Id: 4EPZ) from *Bacteroides uniformis* using ClustalW.^27^ A small segment of this sequence, spontaneously adopting an α helix structure, was integrated into the model built based on the 4IGE structure. Comparative modeling was performed using Modeler 10.1^28^ according to the protocol described by Martí-Renom et al.^29^ One thousand models were generated, and the model with the lowest Discrete Optimized Protein Energy (DOPE) value was selected for validation. The quality of the models was assessed using PROCHECK,^30^ ProSA-web,^31^ QMMEAN,^32^ and MolProbity.^33^

### 2.2 Search and Selection of Candidate Ligands

A comprehensive literature review was conducted to identify triclosan-derived compounds previously investigated for their in vitro activity against PfENR. Studies by Freundlich et al. (2005, 2006, and 2007) and Belluti et al. (2013) were mainly considered, focusing on the IC50 values.^34−37^ Ninety-six ligands were compiled, forming Library 1 (Table S1). The three-dimensional (3D) structures and PfENR-related data for these compounds were obtained from PubChem in *sdf* format.^38^ The Universal Force Field (UFF)^39^ was employed for energy minimization, and the structures were converted to *pdbqt* format using the PyRx 0.9.7 program.^40^

### 2.3 Virtual Screening and Molecular Docking

**Figure 2:**
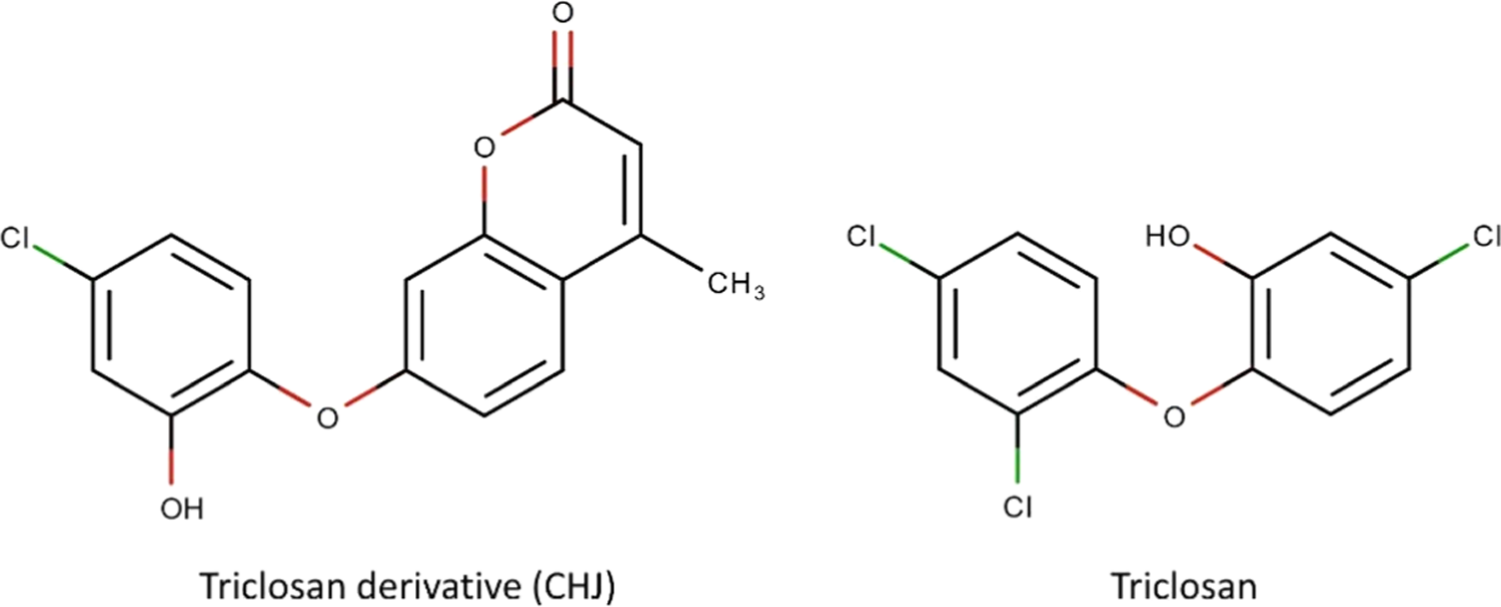
2D schemes of Triclosan and its derivative CHJ.

Rigorous redocking and cross-docking tests were conducted to validate the docking procedure using AutoDock Vina 1.2^41^ and DockThor software^42^ tools with the compound 7-(4-chloro-2-hydroxyphenoxy)-4-methyl-2*H*-chromen-2-one (CHJ—CID: 71768351), derived from Triclosan (TCL) and extracted from PDB 4IGE, used as ligand (Figure 2). Cross-docking was performed by using PDB 3AM5.

To verify if AutoDock Vina 1.2 is able to distinguish active and inactive compounds, a virtual screening using Library 1 was also performed.

The modeled structure was superimposed onto the PDB 4IGE and aligned using Swiss-PDB Viewer v4.1^43^ to determine the docking grid.

A simulation grid centered on the ligand was utilized, with *x*, *y*, and *z* coordinates of 51.328, 91.658, and 34.935 Å, respectively. The box dimensions along the three Cartesian directions were set to 24.061, 20.426, and 15.898 Å with a grid size of 0.375 Å. The input parameters for exhaustiveness and the number of modes were defined as 20 and 9, respectively. The optimal outcomes were determined by minimizing the scoring function, with the cutoff threshold established based on the score attained for the CHJ ligand during the redocking procedure.

A receiver operating characteristic (ROC) curve was generated to assess the discriminatory power of molecular docking in distinguishing between potentially active and inactive compounds and to establish a binding energy threshold for the ligands in Library 1 using Jamovi software, utilizing the Youden index criterion to establish the cutoff point. Active molecules were defined as those with IC50 values of 2.5 μM or less, while inactive compounds were defined as those with IC50 values of 15 μM or more (as shown in Table S1). Ligands falling within the IC50 range of 2.5–15 μM were excluded from the study due to their inherent uncertainty, as evidenced by substantial standard error values reported in the relevant literature.^34−37^ The binding energy of the CHJ ligand was used as an upper limit in the selection process.

The two best-ranked molecules from Library 1 obtained in the virtual screening were used as the lead compound in the compound search of PubChem (September 2022). The search was carried out using the Tanimoto coefficient and generated Library 2 with 335 molecules. The compounds from Library 2 were then subjected to virtual screening using AutoDock Vina 1.2 and DockThor^42^ programs, employing the same input parameters defined earlier. For the DockThor server, the following parameters were used: Number of Evaluations: 500 000, Population Size: 750, Initial Seed: −1985, Number of Runs: 12, with the soft docking option disabled.

To identify the optimal molecular docking results from compounds in Library 2, the score of crystallized ligand CHJ was used as the upper threshold. The results obtained from two different servers were compared, and compounds that demonstrated superior performance in both programs were selected for further analysis and subsequently acquired.

### 2.4 Molecular Dynamics Simulations

Molecular dynamics (MD) simulations were performed using AMBER 22,^44^ and protein interactions were represented using the Amber ff14SB force field.^45^ Bonded, electrostatic, and Lennard–Jones parameters for ligands and NADH cofactor were obtained using the Generalized Amber force field (GAFF)^46^ and AM1-BCC tools.^47^ while atomic partial charges were calculated using ANTECHAMBER.^48^ Electrostatic interactions were treated using the particle mesh Ewald (PME) algorithm with a cutoff of 12 Å. Each system was simulated under periodic boundary conditions in a triclinic box, whose dimensions were automatically defined considering 10 Å from the outermost protein atoms in all Cartesian directions. The simulation box was filled with TIP3P water molecules.^49^

Subsequently, a two-step energy minimization procedure was performed: (i) 2000 steps (1000 steepest descent + 1000 conjugate-gradient) with all heavy atoms harmonically restrained with a force constant of 5 kcal mol^–1^ Å^–2^ and (ii) 5000 steps (2500 steepest descent + 2500 conjugate-gradient) without position restraints. Next, initial atomic velocities were assigned using the Maxwell–Boltzmann distribution corresponding to a temperature of 20 K. Using the Langevin thermostat, the systems were gradually heated up to 300 K over one nanosecond.^50^

All heavy atoms were harmonically restrained during this stage, with a 10 kcal mol^–1^ Å^–2^ force constant. All systems were subsequently equilibrated during nine successive 500 ps equilibration simulations where position restraints approached zero progressively. After this period, all of the systems were simulated with no restraints at 300 K in the Gibbs ensemble with a 1 atm pressure using isotropic coupling. All chemical bonds containing hydrogen atoms were restricted using the SHAKE algorithm,^51^ and the time step was set to 2 fs. Finally, we simulated three independent MD runs of 200 ns for each complex using different initial velocities.

Simulation trajectories were analyzed with GROMACS package tools.^52^ Root-mean-square deviation (RMSD) and root-mean-square fluctuation (RMSF) were calculated separately for each system, fitting their heavy atoms and taking the initial structure of the production dynamics as a reference. Conformational clusterization for a system was performed using the GROMOS method with a cutoff of 2.5 Å, discarding the intrinsically disordered region (IDR). The central structure of the largest cluster was regarded as an additional analysis. Hydrogen bonds (H-bonds) were calculated between protein and ligand complexes. H-bond formation was defined using a geometric criterion with CPPTRAJ^53^ in Amber. We considered a hit when the distance between two polar heavy atoms, with at least one hydrogen atom attached, was less than 3.5 Å and using an H-donor angle higher than 120°.

The enthalpy of PfENR complexes was calculated by extracting the uncorrelated 500 snapshots from each MD simulation’s last 50 ns trajectory (time interval in which the simulations mostly stabilized),^54^ using the MM/GBSA (molecular mechanics generalized Born surface area) approach. All water molecules and counterions were stripped from the trajectories before the MM/GBSA calculation. The interaction energy and solvation-free energy for the complex, receptor (protein and NADH), ligand, and resulting averages were calculated using the MMPBSA.py module^54^ available in the AMBER distribution. Finally, the conformational entropic contribution to the binding free energy was estimated for 50 snapshots using the normal-mode analysis from each MD simulation’s last 50 ns trajectory.

### 2.5 PfENR Expression

PfENR was subcloned into a pGS-21a plasmid and transformed into *E. coli* BL21(DE3) cells, as described by Medeiros and colleagues (2011).^55^ The transformation step was carried out with the BL21(DE3) strain of *E. coli* (DE3) bacteria by electroporation (MicroPulser, Bio-Rad, EUA) with a pulse of 1.8 kV with an average duration between 4.9 and 5.3 ms and plated on gar Luria–Bertani (LB) (Invitrogen, EUA) containing ampicillin (100 μg/mL) (LB/Amp). For recombinant PfENR expression, isolated colonies were grown in an Erlenmeyer flask containing 5 mL of Luria–Bertani (LB) medium with 100 μg/mL ampicillin and incubated under shaking conditions (200 rpm) at 37 °C for 16 h. After this period, about 2% of the preinoculum volume was transferred to 50 mL of LB/Amp (100 μg/mL ampicillin) and maintained under agitation (200 rpm) at 37 °C until reaching an optical density of 0.8 in a waveform length of 600 nm (DO_600_). To induce recombinant PfENR expression, isopropyl-*d*-1-thiogalactopyranoside (IPTG) was added at a final concentration of 1 mM, and the culture was grown under agitation (200 rpm) for 12 h at 37 °C.

The Oswaldo Cruz Rondônia Foundation (Fiocruz-RO) possesses a certificate for the manipulation of genetically modified organisms (GMOs) under registration CQB no 391/15, with an extension to the Laboratory of Bioinformatics and Medicinal Chemistry.

### 2.6 PfENR Purification

The bacterial culture was centrifuged at 4500 rpm for 15 min at 4 °C. The bacteria pellet was resuspended in a lysis buffer solution (Tris/HCl 50 mM with Triton X-100 0.5%, pH 8.0) and subjected to cell lysis in a sonicator (amplitude 40, pulse time 15 s, rest time 45 s, sonicating for 4 min) in an ice bath. The material was centrifuged at 10 500 rpm at 4 °C for 15 min. For the purification of PfENR, fused with a histidine tag (HIS), affinity chromatography on Ni^2+^ (QIAGEN) was performed. The equilibration buffer was 50 mM Tris, 300 mM NaCl, and 20 mM Imidazole, pH 8.0, and the elution buffer was 50 mM Tris, 300 mM NaCl, and 250 mM Imidazole, pH 8.0. The collected fractions were subjected to sodium dodecyl sulfate-poly(acrylamide gel) electrophoresis 12% (SDS-PAGE 12%) analysis. C18 reversed-phase chromatography was performed for better purification, with the peak eluted in Ni^2+^ affinity chromatography. The equilibration solution was 0.1% trifluoroacetic acid (0.1% TFA), and the elution solution was acetonitrile with 0.1% TFA (ACN, 0.1% TFA).

### 2.7 PfENR Inhibition Assay

To evaluate the IC50 of lead compounds TCL and LG2 (Ligand 2), NADH consumption was measured at varying concentrations in triplicate, as described by Lindert and colleagues (2015).^56^ The final 200 μL reaction volume contained 20 mM Tris/HCl buffer, 150 mM NaCl (pH 7.4), 240 μM crotonyl-CoA, 100 μM NADH, and 0.05 μM PfENR. PfENR was preincubated at 28 °C for 5 min with a 0.02–80 μM inhibitor. Following this preincubation period, the reaction was initiated with a final concentration of 240 μM crotonyl-CoA, 20 mM Tris/HCl buffer, 150 mM NaCl (pH 7.4), and 100 μM NADH. The IC50 values, representing the inhibitor concentrations at which 50% inhibition was observed, were determined using GraphPad Prism software. A nonlinear regression analysis was used to obtain these values.

## 3 Results and Discussion

### 3.1 Modeling the PfENR 3D Structure

The search for 3D structures of PfENR in the PDB using the canonical sequence (NCBI XP_966137) returned several results. Structural analysis of PfENR based on the available 3D structures in the Protein Data Bank (PDB) revealed the presence of a structurally unresolved region within the protein, suggesting the possibility of an intrinsically disordered region (IDR). Computational techniques widely used for predicting intrinsically disordered regions were employed to investigate these regions in the PfENR protein sequence.^57,58^

Analyses of the results identified two regions, specifically residues 45–60 and 235–270, that displayed a high probability of being intrinsically disordered (Figure S1A). Notably, the region encompassing residues 45–60 in the PfENR structure 4IGE corresponded to a partial α helix. Conversely, residues 235–270 were not observed in the available PfENR structures,^37^ providing further support for the presence of an IDR in this region. Secondary structure prediction using PSIPRED and the JPred protein also indicated a coil conformation for this region, except for a short α helix segment defined by residues 261–264 (–EEKK–) (Figure S1B). It is worth noting that this α helix segment demonstrated a piDDT value below 50, as determined by the AlphaFold algorithm.^59^

**Figure 3:**
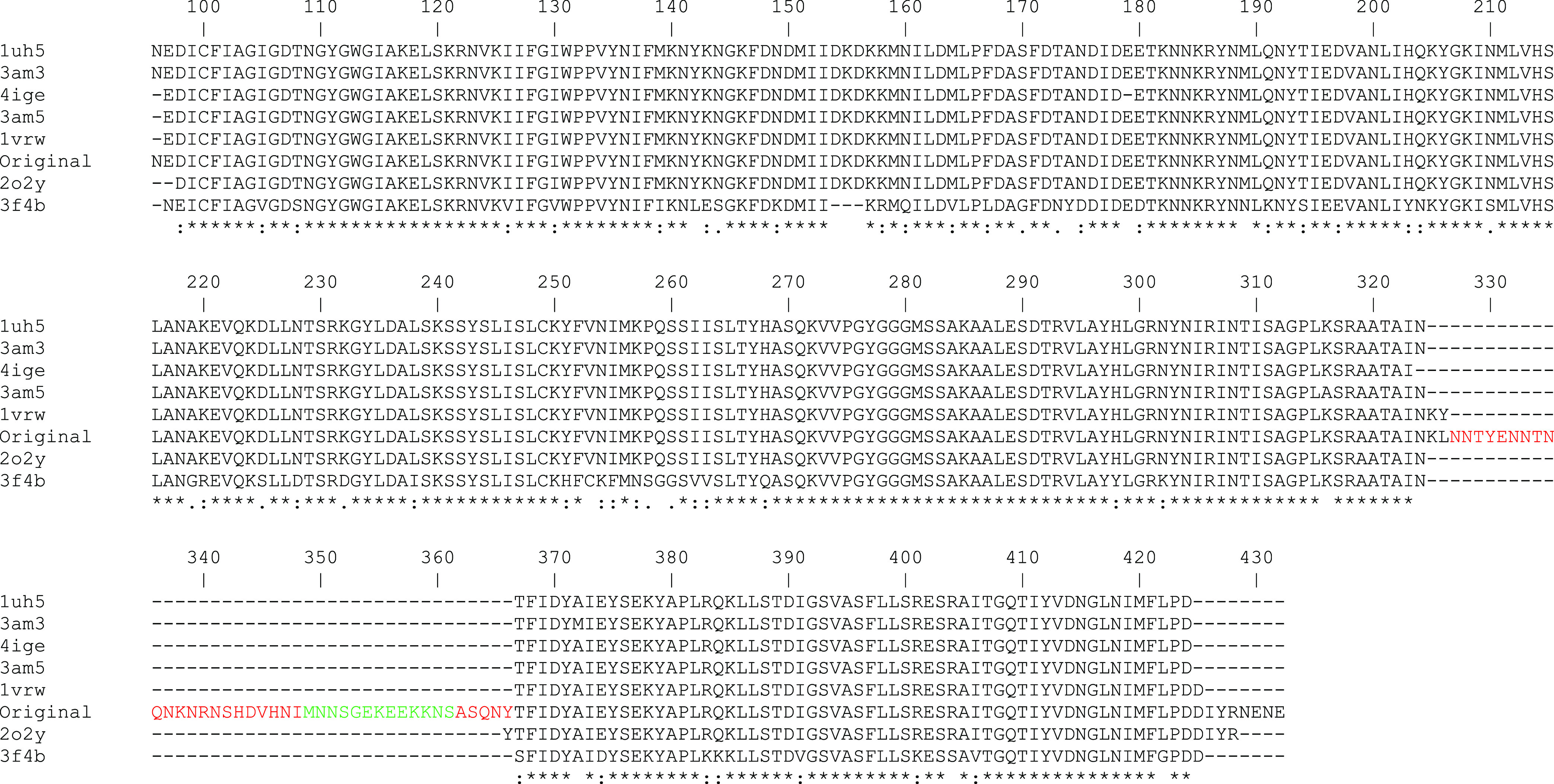
Sequence alignment of PfENR (original) with other PfENR sequences obtained from the PDB archives (PDB codes: 1uh5, 3am3, 4ige, 3am5, 1vrw, 2o2y, and 3f4b). The alignment identified a region (amino acids 320–370) with an unresolved secondary structure. Coil regions are highlighted in red, while modeled regions are depicted in green.

The structure under PDB 4IGE was selected for its greater coverage and resolution. However, no structure was observed between positions 261–264, which were modeled by homology. To model the missing structure within the IDR of PfENR, a Blast query was performed against the PDB, targeting residues 232–271. A crystal structure (PDB code: 4EPZ) corresponding to a transcription antiterminator antagonist from *B. uniformis* was found, with a 39% sequence similarity with the target sequence. This *B. uniformis* structure was selected as a suitable template for modeling the missing region in PfENR (Figure 3).

The modeled structure was subjected to rigorous validation, and the analyses confirmed that over 90 percent of the amino acids in the model resided in favorable regions, with no residues falling into the disallowed region of the Ramachandran plot (Figure S2). The geometric and stereochemical analyses of the Ramachandran plot revealed a favorable structural quality, with approximately 90.3% of the amino acids occupying preferred regions. The ProSA-web *Z*-score, calculated to assess model quality against native structures, yielded a value of −6.77, signifying strong statistical compatibility (Figure S3A). Moreover, MolProbity analysis confirmed the phi and psi angle data obtained from PROCHECK and detected no issues with covalent bonds or anomalous C-α geometry.

The QMEAN method was employed to evaluate the structural quality of the generated model of PfENR, considering its physicochemical properties. This approach produced a QMEAN score of −1.60 for the model compared to the QMEAN scores of 9766 high-resolution experimental structures.^32^ Remarkably, the QMEAN score of the PfENR structural model closely resembled that of the high-resolution experimental structures, as illustrated in Figure S3B. Furthermore, the root-mean-square deviation (RMSD) between the modeled structure and the template (PDB code: 4IGE) was determined to be 0.218 Å.

### 3.2 Library 1 Composition and PfENR Structure Selection

To investigate the triclosan derivatives and their potential as inhibitors of PfENR, Library 1 was constructed by extensively reviewing four seminal publications that focused on modifying the triclosan scaffold with various substituents.^34−37^ These modifications introduced diverse functional groups, including nitrile, hydroxyl, amide derivatives, aniline, and others, thereby expanding the chemical versatility of the triclosan core. This strategy of exploring compounds with similar but untested structures based on molecules previously evaluated against a specific target is a standard approach in this field.^40,60,61^ The complete list of selected compounds in Library 1 can be found in Table S1.

Before the molecular docking experiments were conducted, an extensive search was conducted to acquire the three-dimensional structures of PfENR. The Protein Data Bank (PDB) yielded nine structures that met the criteria (Table S2), providing a comprehensive set of options for analysis. Among these structures, 4IGE, despite having a slightly higher resolution (2.15 Å), was selected as the primary candidate for the molecular docking tests due to its remarkable 100% similarity to the target enzyme. The ligand complexed with structure 4IGE is a derivative of triclosan, specifically 7-(4-chloro-2-hydroxyphenoxy)-4-methyl-2*H*-chromen-2-one (CHJ).

### 3.3 Virtual Screening and Molecular Docking

To validate the docking protocol, redocking and cross-docking experiments were performed. Redocking of the CHJ ligand into the 4IGE binding site resulted in an RMSD of 0.67 Å, while cross-docking experiments using ligands from the 4IGE and 3AM5 structures yielded RMSDs of 0.91 and 0.51 Å, respectively. These results indicate the reliability and accuracy of the molecular docking approach.

**Figure 4:**
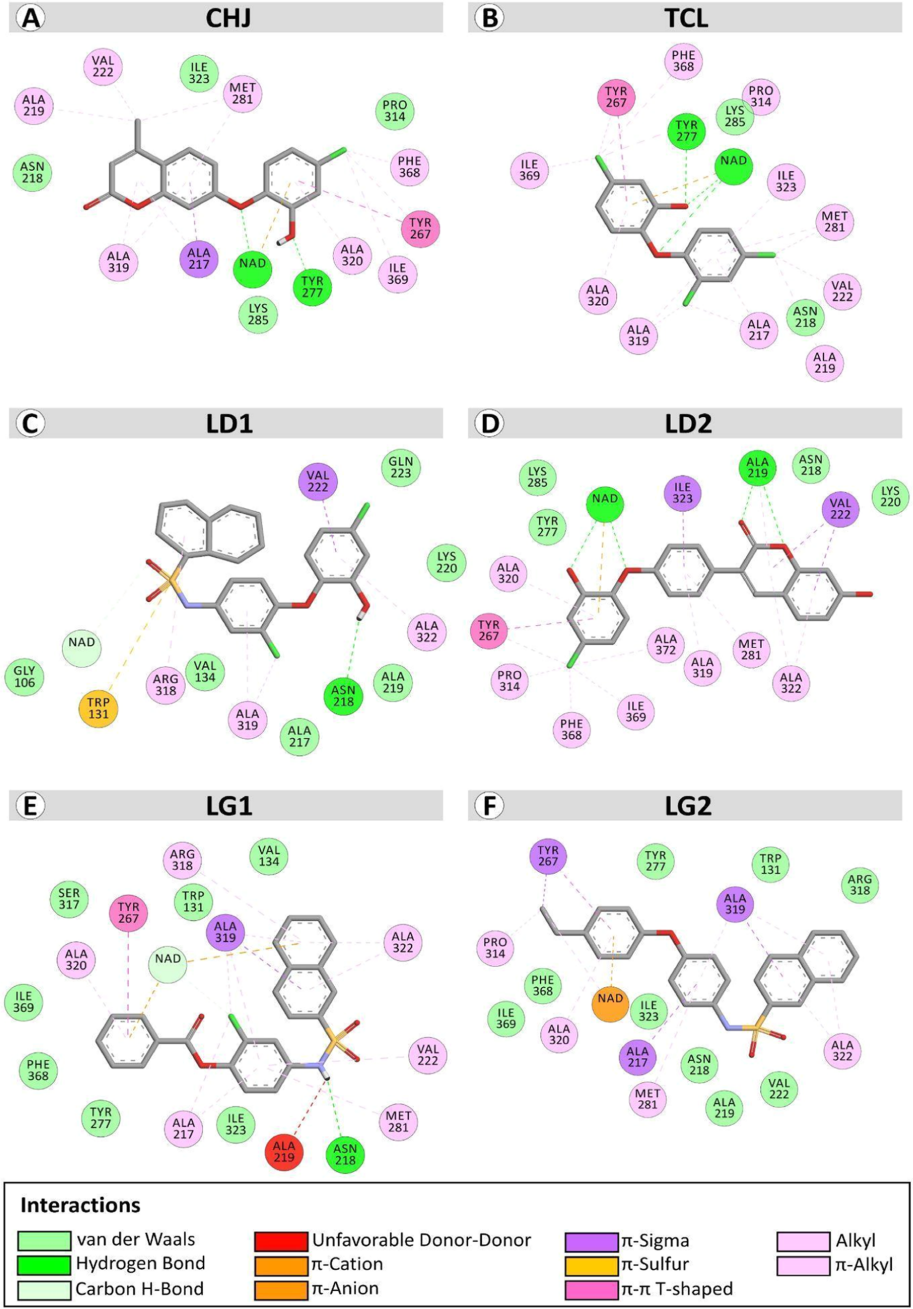
Two-dimensional schemes extracted from the molecular docking of interactions between the active site region of PfENR and the ligands: (A) CID: 71768351 (CHJ); (B) Triclosan; (C) CID: 44405336 (LD1); (D) CID: 72703246 (LD2); (E) 4245155 (Ligand 1—LG1); and (F) Ligand 2—LG2*. The images were generated by using BIOVIA Discovery Studio Visualizer 21.1.0.0.

To identify potential lead compounds for inhibiting PfENR, virtual screening and molecular docking were performed using ligands from Library 1. The Vina scoring function was used to evaluate the affinity of the ligands, and seven compounds were found to have lower binding energies than both the crystallographic CHJ ligand (ICD: 71768351, Δ*G* = −10.00 kcal/mol, Figure 4A) and the standard ligand TCL (ICD: 5564, Δ*G* = −9.20 kcal/mol, Figure 4B) (Table S3). Among these, LD1 (CID: 44405336, lead compounds 1) with a binding energy of Δ*G* = −11.0 kcal/mol (Figure 4C) and LD2 (CID: 72703246, lead compounds 2) with a binding energy of Δ*G* = −10.9 kcal/mol (Figure 4D) displayed the lowest binding energies and were selected as lead compounds for further investigation.

The standard ligands TCL and CHJ were selected as positive controls based on previous evaluations in in silico and in vitro studies against PfENR. The interactions between TCL and CHJ primarily involve essential hydrogen bonding interactions with Tyr277 and the cofactor NAD^+^, accompanied by additional electrostatic interactions such as pi-cation interactions with NAD^+^. Both ligands also engage in hydrophobic interactions, contributing to their binding affinity.^37,57,60^ In contrast, distinct interaction patterns were observed when the interactions of lead compounds LD1 and LD2 with the reference ligands TCL and CHJ at the PfENR binding site. In this case, LD1 and LD2 exhibited different predominant interactions, particularly in hydrogen bonding and electrostatic interactions, compared with the standard ligands TCL and CHJ.

The results obtained from previous in vitro investigations showed that LD1 and LD2 exhibited IC50 values in the micromolar scale (2.5 and 0.45 μM, respectively),^35,36^ supporting their selection as primary compounds for this study. Subsequently, molecular docking results from this work revealed substantially improved binding energies for LD1 and LD2, measuring −11.0 and −10.9 kcal/mol, respectively.

Ligand 1 from Library 2 exhibited interactions similar to those observed for LD1, involving hydrogen bonds with Asp218. In contrast, Ligand 2 did not form hydrogen bonds with PfENR. Ligands LG1 and LG2 underwent molecular dynamics analysis to obtain further information about their stability and will also be subjected to in vitro analyses to verify their inhibitory potential against PfENR.

**Figure 5:**
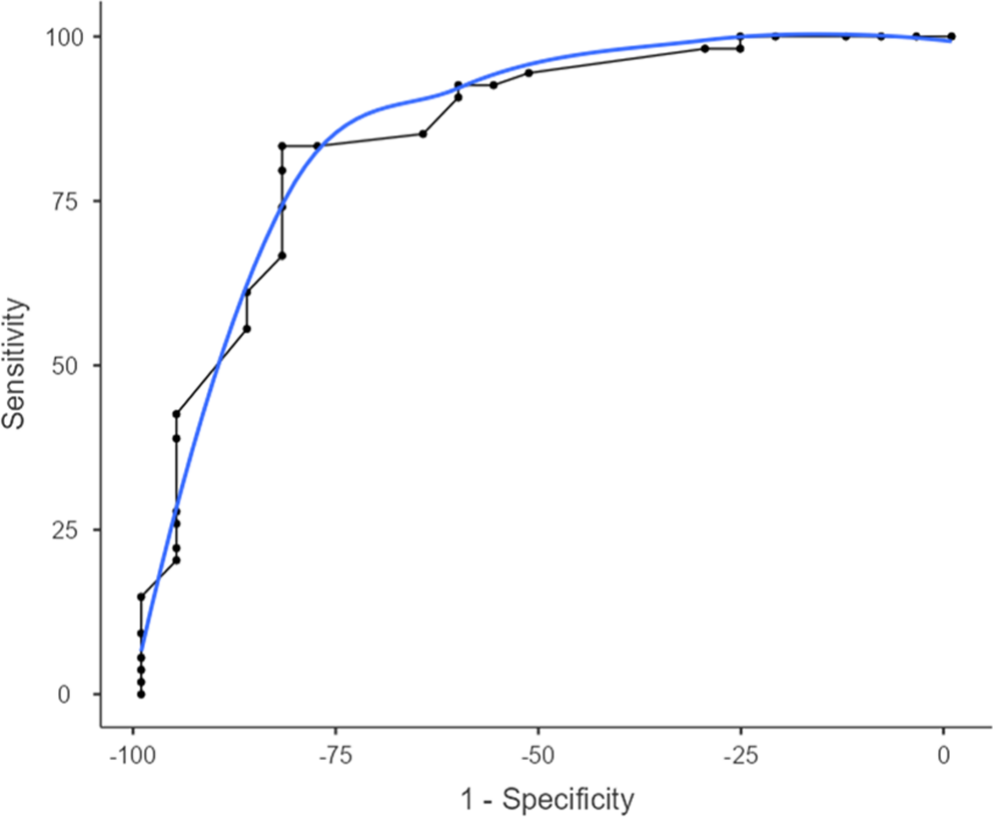
ROC curve illustrating the correlation between the energy values obtained from molecular docking and the in vitro inhibition results of the tested ligands against PfENR. The area under the curve (AUC) value was determined to be 85.6%.

The receiver operating characteristic (ROC) curve was generated to assess the discriminatory power of the molecular docking approach in distinguishing between active and inactive ligands from Library 1 against PfENR. Following de Oliveira et al. (2022),^62^ the AUC values derived from the ROC curve were used to distinguish between false positives and true positives. Ligands with known in vitro inhibitory activity were regarded as true positives (TP), while compounds lacking inhibitory activity were classified as true negatives (TN). The ROC curve exhibited an area under the curve (AUC) value of 0.856, indicating a high level of accuracy and an 85.6% probability of correct classification (Figure 5).

Meticulous ROC curve analysis established a critical threshold based on binding energy. Specifically, a cutoff value of −9.10 kcal/mol was determined, signifying the point at which the best metric score was achieved, aligning with other robust evaluation criteria to define the threshold (Table 1). Ligands exhibiting binding energies below this critical threshold are classified as active, demonstrating strong potential for PfENR inhibition, while those exceeding this threshold are considered inactive.

**Table 1:** Cutoff Points, Corresponding Sensitivity, Specificity, and Metric Score Values Derived from Molecular Docking Data and Activity Profiles of Ligands in Library 1

cutpoint (kcal/mol)	sensitivity (%)	specificity (%)	youden’s index	metric Score
–9.0	83.64%	78.26%	0.619	1.62
–9.1	83.64%	82.61%	0.662	1.66
–9.2	80%	82.61%	0.626	1.63

The calculation of the cutoff point on the ROC curve was based on the Youden index method. The Youden index is the maximum vertical distance between the ROC curve and the diagonal line connecting the points (0,0) and (1,1). The equation is *J* = sensitivity + specificity 1, which is applied to each point on the ROC curve. By using the Youden Index, Jamovi helps find the threshold that balances the sensitivity and specificity, maximizing the overall classification accuracy of the model.

Despite the energy given by the Youden index (−9.1 kcal/mol), which may be deemed to be a promising threshold, a more rigorous criterion was used as an upper limit. Instead, we used the binding energy of the CHJ ligand (−10.00 kcal/mol) calculated during redocking in VINA. Using this threshold, LD1 and LD2 were selected as lead compounds for further virtual screening. They were then screened against the PubChem/NCBI ligand database using the Tanimoto index as a similarity filter. LD1 yielded 187 molecules with significant similarity at a threshold of 78%, while LD2 resulted in 148 similar molecules at an 86% similarity threshold. These compounds were compiled to form Library 2, consisting of 335 ligands, which would be evaluated against PfENR in subsequent analyses.

The decision to use only LD1 and LD2, despite the availability of other options, was based on the criterion of selecting those that exhibited superior binding energy. This underscores the importance of optimizing ligand selection to ensure more effective interaction between molecules, ultimately yielding more promising results in research for the discovery of new, specific compounds for PfENR.

Library 2 ligands underwent evaluation using AutoDock Vina and DockThor, with the CHJ binding energy being at the threshold. Among the ligands, 18 compounds exhibited lower binding energies than CHJ in both programs, justifying their selection for further analysis (Table 2). Notably, these selected molecules demonstrated binding energies superior to those of CHJ in both docking programs. One ligand, LG1 (CID: 4245155) (Figure 4E), showed promising results in both AutoDock Vina (Δ*G* = −11.5 kcal/mol, Top 1) and DockThor (Δ*G* = −9.311 kcal/mol, Top 27). Therefore, LG1 was commercially acquired for subsequent in vitro experiments. Unfortunately, the product corresponding to the binder CID: 3666133, which displayed the second-best result in AutoDock Vina, was unavailable. Instead, an alternative binder denoted as LG2 was provided. LG2 exhibited a substitution of a methyl group with an ethyl group at position 4 of the phenoxy moiety, thereby yielding compound *N*-[4-(4-ethylphenoxy)phenyl]naphthalene-2-sulfonamide. In AutoDock Vina, LG2 exhibited a binding energy of −10.8 kcal/mol (Figure 4F). The remaining binders listed in Table 2 were either unavailable for commercial purchase or were currently in the quotation process.

**Table 2:** Molecular Docking Outcomes of Library 2 Ligands Outperformed the Reference Value of CHJ Using Autodock Vina and DockThor Programs

	binding energy (kcal/mol)	
CID	autodock vina	dockThor
3666133	–11.2	–9.422
7938763	–10.9	–9.348
93734272	–10.8	–9.843
58565630	–10.8	–9.555
1370637	–10.7	–9.450
56727544	–10.7	–9.392
44405289	–10.6	–9.544
93734284	–10.4	–9.240
93734285	–10.4	–10.025
15952563	–10.4	–9.271
52100125	–10.4	–9.409
14673679	–10.3	–9.679
1217801	–10.3	–9.261
3546524	–10.3	–9.398
3782585	–10.3	–9.394
1217824	–10.2	–9.606
139940601	–10.1	–9.259
CHJ	–10.0	–9.238

A comprehensive analysis of ligand interactions, including reference ligands CHJ and TCL, as well as lead ligands LD1 and LD2 from molecular docking of Library 1 and Library 2 ligands, and commercially purchased ligands LG1 and LG2, was conducted (Table S4).

The reference ligands TCL and CHJ were selected as positive controls due to their previous evaluation in silico and in vitro studies against PfENR. Interactions between TCL and CHJ primarily involve fundamental hydrogen bonding interactions with Tyr277 and the cofactor NAD+, accompanied by additional p-cation-type electrostatic interactions with NAD+. Both ligands also engage in hydrophobic interactions, contributing to their binding affinity.^37,57,60^ In contrast, distinct interaction patterns were observed when comparing the lead compound LD1 and LD2 interactions with the TCL and CHJ reference ligands within the PfENR binding site. Notably, LD1 and LD2 exhibited different predominant interactions, particularly in hydrogen bonding and electrostatic interactions, compared to those of the TCL and CHJ standard ligands.

The outcomes obtained in previous in vitro investigations demonstrated that LD1 and LD2 displayed micromolar IC50 values (2.5 and 0.45 μM, respectively),^34,37^ thus supporting their selection as the primary compounds for this study. Subsequently, the molecular docking results from our ongoing investigation revealed substantially improved binding energies for LD1 and LD2, measuring −11.0 and −10.9 kcal/mol, respectively.

Ligand 1 from Library 2 exhibited interactions similar to those observed for LD1, involving hydrogen bonding with Asp218. In contrast, Ligand 2 did not form hydrogen bonds with the receptor. These outcomes strongly imply robust binding affinities between LD1 and LD2, and PfENR, thereby underscoring their potential as promising inhibitors. LG1 and LG2 underwent molecular dynamics analysis to gain further insights into their stability, which will provide valuable information for subsequent in vitro experimentation, supporting their potential as PfENR inhibitors.

### 3.4 Molecular Dynamics Simulations

Molecular dynamics (MD) simulations have practical applications in refining docking results, validating ligand placement, and exploring critical interactions to evaluate the stability and reliability of docked positions.^63^ Practical MD simulations can differentiate between correct positions and false positives, as demonstrated by Liu et al., where erroneous poses exhibited an RMSD greater than 2 Å and ligands dissociated from the binding site within the initial nanoseconds.^64^

**Figure 6:**
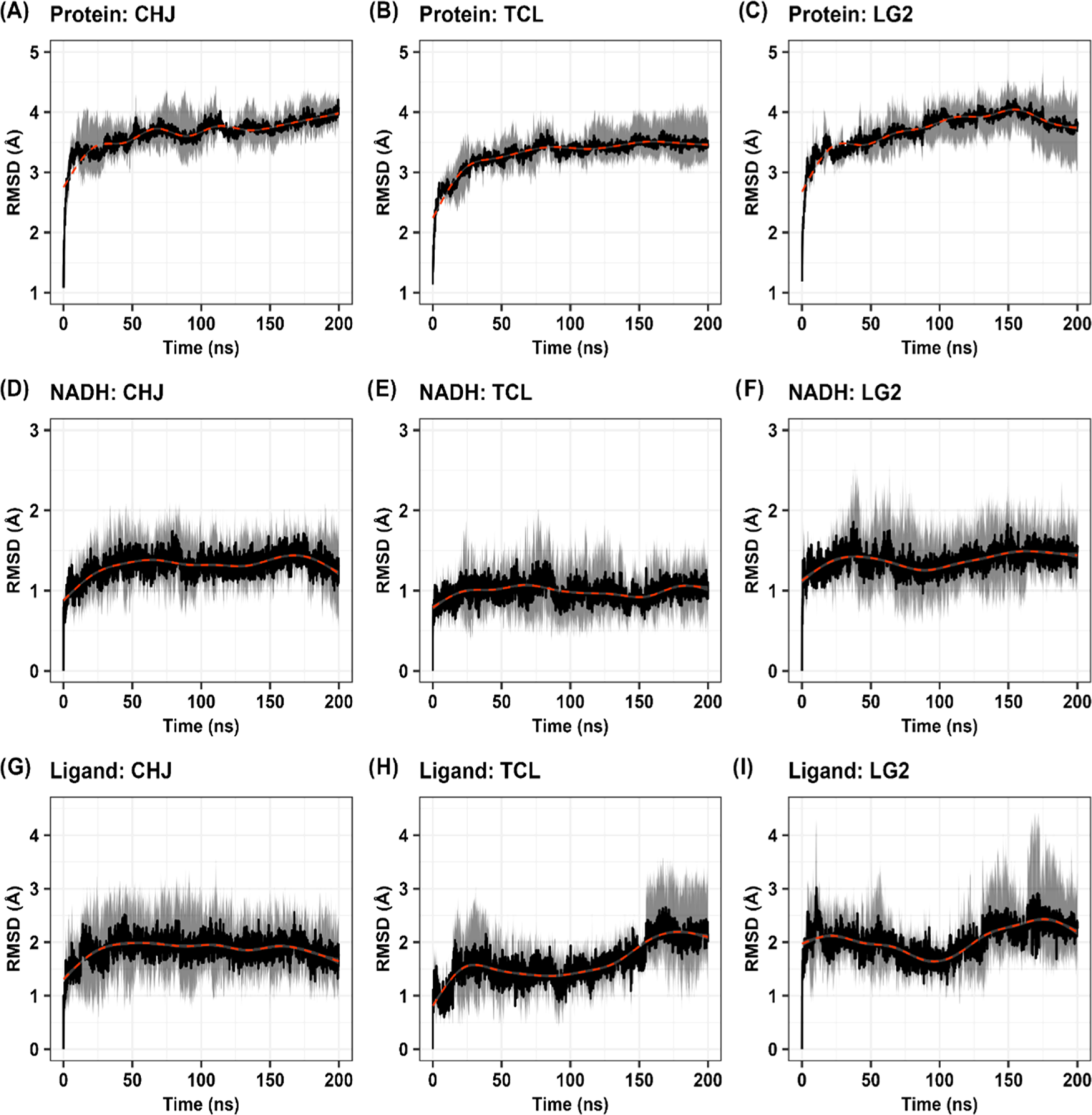
Root-mean-square deviation (RMSD) profiles obtained from molecular simulations of the Protein-NADH-Ligand systems. Each plot corresponds to a specific system: CHJ (A, D, and G), TCL (B, E, and H), and LG2 (C, F, and I). The black line represents the mean of triplicate simulations, and the gray area represents the confidence interval. The red dashed line denotes the trend line.

In our MD simulations, the LG1 pose was lost within the first 50 ns, leading to disrupted interactions and the subsequent exit of the ligand from the binding site (Figure S4). In contrast, the positions of the other ligands remained stable at the binding site. Among the systems, TCL exhibited the greater stability, as evidenced by the lower RMSD values compared to CHJ and LG2, with values of 1.62 ± 0.62, 1.86 ± 0.62, and 2.05 ± 0.63 Å, respectively (Figure 6G–I). The RMSD values of the cofactor NADH in the simulations involving CHJ, TCL, and LG2 were 1.31 ± 0.33, 0.99 ± 0.33, and 1.37 ± 0.38 Å, respectively (Figure 6D–F). The RMSD values of the protein ranged from 3.31 ± 0.38 Å (in TCL) to 3.70 ± 0.42 Å (in LG2), with a negligible difference below 0.4 Å (Figure 6A–C). The overall fluctuation pattern was similar among all systems, with the highest peaks observed in the intrinsically disordered region (IDR) (Figure S5).

Calculating binding free energy is crucial for understanding ligand association and recognition, allowing for ranking drug candidates based on their binding affinities.^65^ This study employed the MM/GBSA method to determine the absolute binding free energy (Δ*G*_bind_) of the CHJ, TCL, and LG2 compounds to PfENR.^66^ Our results showed negative Δ*G*_bind_ values for all systems (Table 3). The CHJ and TCL systems displayed comparable binding affinities of −15.14 ± 1.27 and −15.24 ± 0.87 kcal/mol, respectively, while LG2 exhibited a more pronounced difference in the binding free energy, with a Δ*G*_bind_ of −19.58 ± 0.94 kcal/mol.

**Table 3:** Binding Free Energies for Complexes Calculated by the MM/GBSA Method^a^,^d^

items	CHJ	TCL	LG2
Δ*E*_vdw_	–48.48 ± 0.093	–41.51 ± 0.085	–58.85 ± 0.194
Δ*E*_ele_	–15.55 ± 0.139	–9.44 ± 0.183	–18.85 ± 0.185
Δ*G*_egb_	30.91 ± 0.154	22.75 ± 0.174	39.55 ± 0.142
Δ*G*_esurf_	–5.16 ± 0.004	–4.51 ± 0.006	–6.98 ± 0.017
bΔ*G*_ele+egb_	15.36 ± 0.146	13.31 ± 0.178	20.7 ± 0.163
cΔ*H*	–38.28 ± 0.102	–32.72 ± 0.088	–45.14 ± 0.204
–*T*Δ*S*	–23.14 ± 1.27	–17.48 ± 0.87	–25.56 ± 0.92
Δ*G*_bind_	**–**15.14 ± 1.27	–15.24 ± 0.87	–19.58 ± 0.94

a All mean and standard error values are given in kcal/mol.

b Δ*G*_ele+egb_ = Δ*E*_ele_ + Δ*G*_egb_.

c Δ*H* = Δ*E*_vdw_ + Δ*E*_ele_ + Δ*G*_esurf_ + Δ*G*_egb_.

d *T* = 298.15 K.

**Figure 7:**
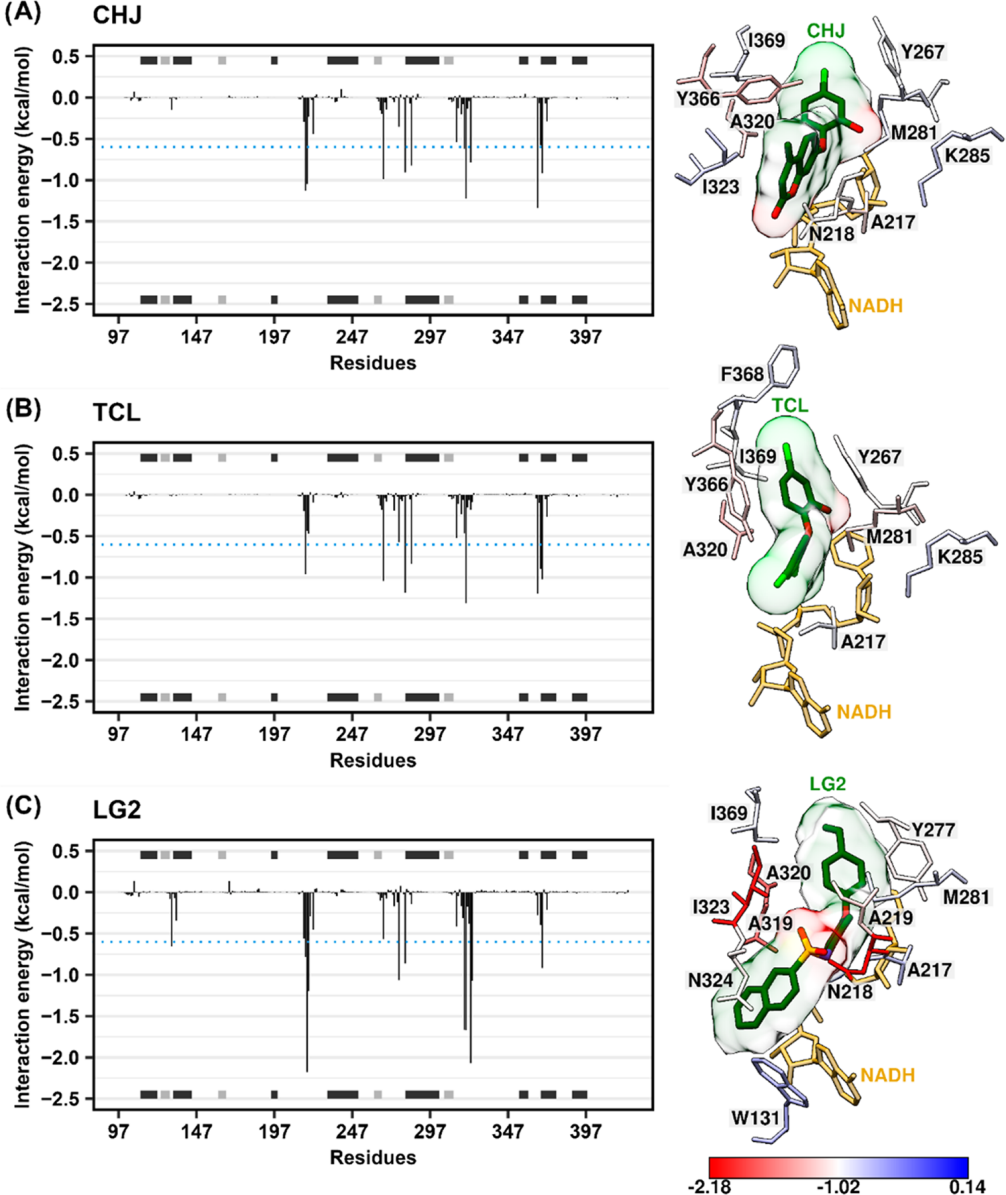
Decomposition of the binding free energy for the simulated systems: CHJ (A), TCL (B), and LG2 (C), bound to the active site of PfENR. The graph illustrates the contributions of individual residues to the ligand–protein interactions. The blue dashed line represents a cutoff of −0.6 kcal/mol, indicating residues with significant contributions. The interaction scale in the lower right corner highlights the residues with the highest contribution, ranging from red (−2.18 kcal/mol) to blue (0.14 kcal/mol).

We further analyzed the contribution of binding site residues to the total binding energy by using the MM/GBSA method. Residues Ala217, Met281, Ala320, and Iso369 consistently exhibited favorable energy values below −0.78 kcal/mol in all complexes (Figure 7). Tyr267, Lys285, and Tyr366 showed energy values lower than −0.6 kcal/mol exclusively in CHJ and TCL complexes (Figure 7A,B). LG2, on the other hand, showed interactions with Trp131, Ala219, Tyr277, Ala319, and Asn324 (Figure 7C).

**Figure 8:**
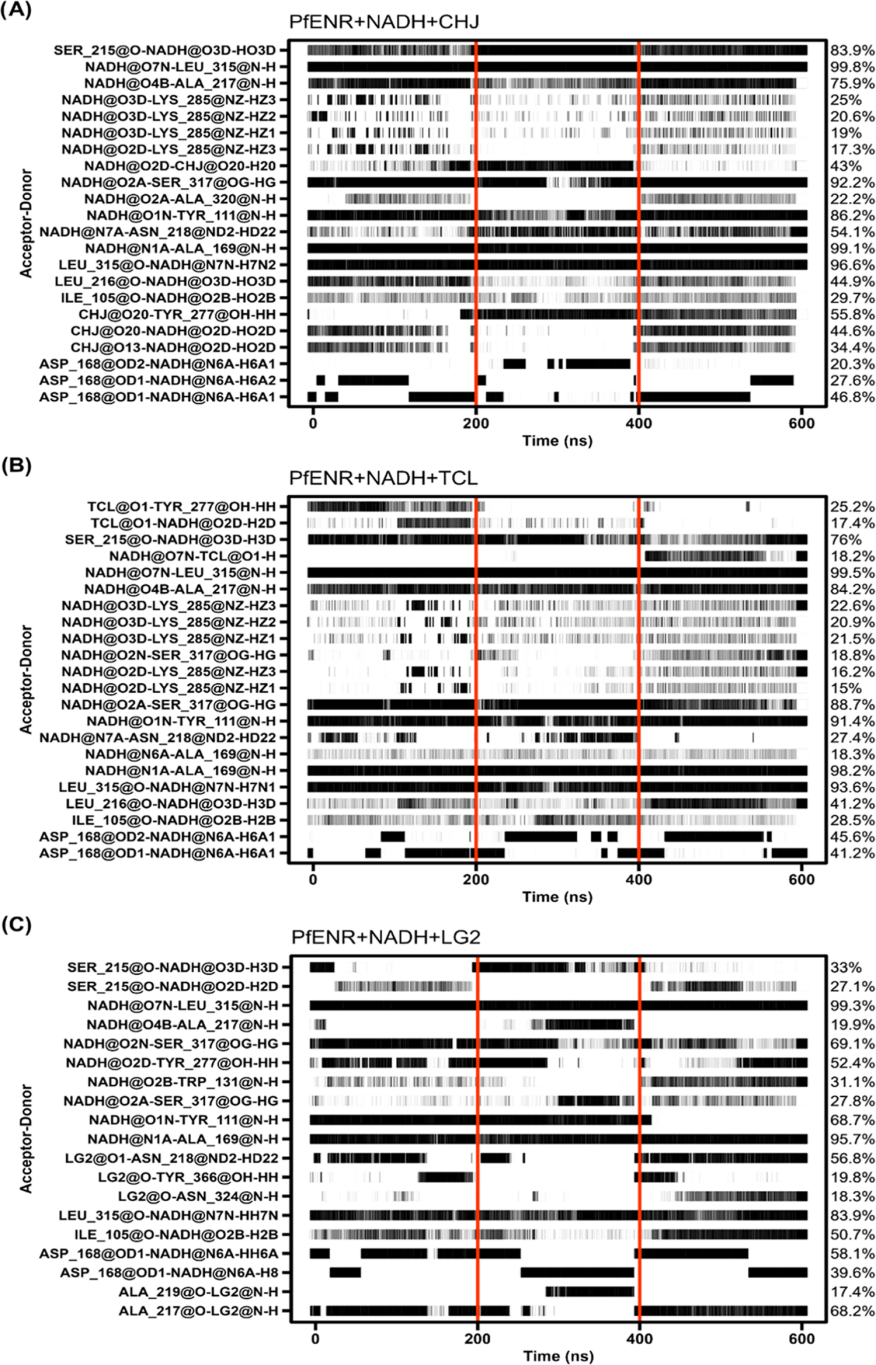
Hydrogen bonding interactions between PfENR and the ligands NADH–CHJ (A), NADH-LG1 (B), and NADH-LG2 (C). The occupancy of each hydrogen bond is indicated on the right axis. The calculations were performed for the final 200 ns of each replica, as denoted by the red vertical lines.

It is worth noting that Tyr277, a catalytic residue, has been reported as an essential amino acid involved in the inhibition of PfENR by triclosan derivatives through hydrogen bond interactions (H-bond).^57,60^ Our analysis revealed that Tyr277 formed H-bonds with CHJ and TCL, with occupancies of 55.8 and 25.2%, respectively (Figure 8A,B), although these interactions made a relatively modest contribution to the overall binding energy (−0.35 kcal/mol for CHJ and −0.57 kcal/mol for TCL). Interestingly, LG2 did not form a hydrogen bond with Tyr277 (Figure 8C) but contributed −1.06 kcal/mol to the binding energy. Instead, LG2 formed an H-bond with Asn218, which exhibited the lowest energy contribution among all systems, amounting to −2.17 kcal/mol. The dynamic analysis does not contain the same richness of information about interactions as docking analysis software (Alkyl, Pi-Alkyl). There is currently no software capable of performing these analyses during a dynamic simulation. However, an analysis of energy decomposition during dynamics is sufficient to associate which amino acids are contributing more or less during the simulation. These interactions are described in the software manual as van der Waals, Electrostatic, Polar Solvation, and Non-Polar Solvation. These findings provide crucial insights into the potential interactions between inhibitors and the PfENR enzyme, shedding light on these interactions’ relative strength and significance.

This study shows that the molecules tested against PfENR do not adhere strictly to the predefined interaction patterns used during the initial search. Notably, ligands LG1 and LG2 exhibit distinct interaction profiles compared with the control compounds TCL and CHJ. However, LG2 shows superior binding energy in molecular docking and demonstrates more robust interactions throughout the molecular dynamics trajectories. Consequently, both LG1 and LG2 were acquired commercially for further investigation.

### 3.5 PfENR Expression and Purification

PfENR, a well-established target for antimalarial interventions, was successfully expressed in a microbial system.^67,68^ The use of the *E. coli* BL21(DE3) strain for heterologous expression of PfENR has been extensively documented in the scientific literature. This strain carries the RNA polymerase gene derived from the T7 phage under control to the IPTG-inducible promoter, making it compatible with any expression plasmid harboring the T7 promoter and providing an efficient platform for PfENR protein production.^16,69−71^

**Figure 9:**
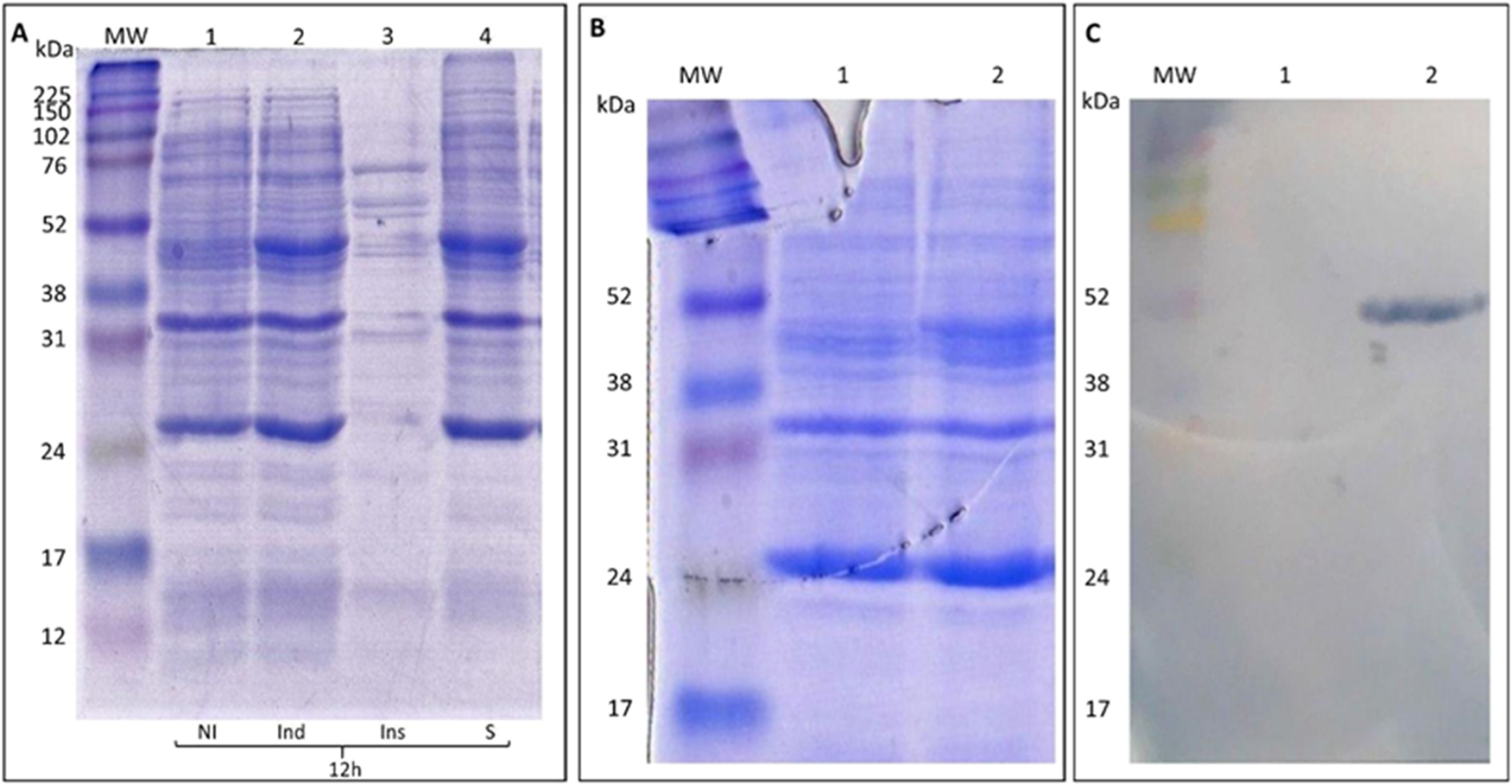
(A) 12% SDS-PAGE of the induction period at 12 h. Lane description: MW: molecular weight standard; (1) uninduced total lysate (NI); (2) induced total lysate (Ind); (3) the insoluble fraction obtained from induced cell lysis (Ins); and (4) the soluble fraction is obtained from induced cell lysis (S). (B) The 12% SDS-PAGE mirror image for Western blot: MW: molecular weight in kDa; (1) 12 h uninduced cells; and (2) 12 h induced cells. (C) Western blot on the nitrocellulose membrane: MW: molecular weight in kDa; (1) 12 h uninduced cells and (2) 12 h induced cells.

The expression of PfENR/pGS-21a in *E. coli* BL21(DE3) was analyzed using poly(acrylamide gel electrophoresis). The gel revealed a prominent band at approximately 50 kDa, indicating significant expression of the PfENR protein (Figure 9A). An anti-his tag Western blot analysis was performed to confirm the presence of PfENR with a polyhistidine tag in the expressed material. A 12% SDS-PAGE electrophoresis was conducted on the uninduced (lane 1) and induced (lane 2) cultures, as shown in Figure 9B. The corresponding Western blot (Figure 9C) demonstrated the presence of PfENR exclusively in the induced material, validating the successful expression of PfENR with the expected polyhistidine tag.

**Figure 10:**
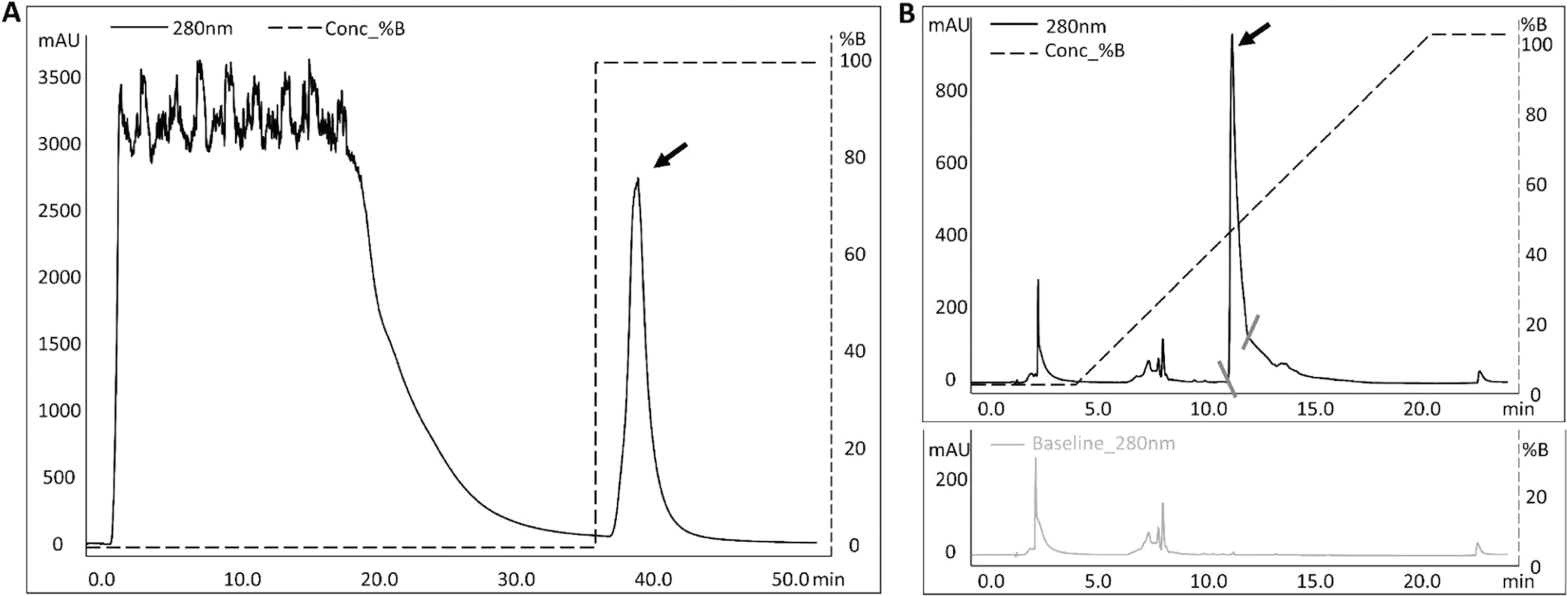
Purification of PfENR. (A) Ni^2+^ affinity chromatography and (B) reversed-phase chromatography on C18.

The elution profile obtained from the purification process showed the appearance of PfENR during the elution phase, spanning from 37 to 43 min, with an intensity level of approximately 2750 mAU (Figure 10A). Subsequently, the collected fraction underwent reversed-phase chromatography (C18) to enhance its purity. A blank analysis was performed during reversed-phase chromatography to establish the baseline. Notably, PfENR (peak 1) eluted at approximately 11.5 min with a composition of approximately 43% eluent B (Figure 10B).

**Figure 11:**
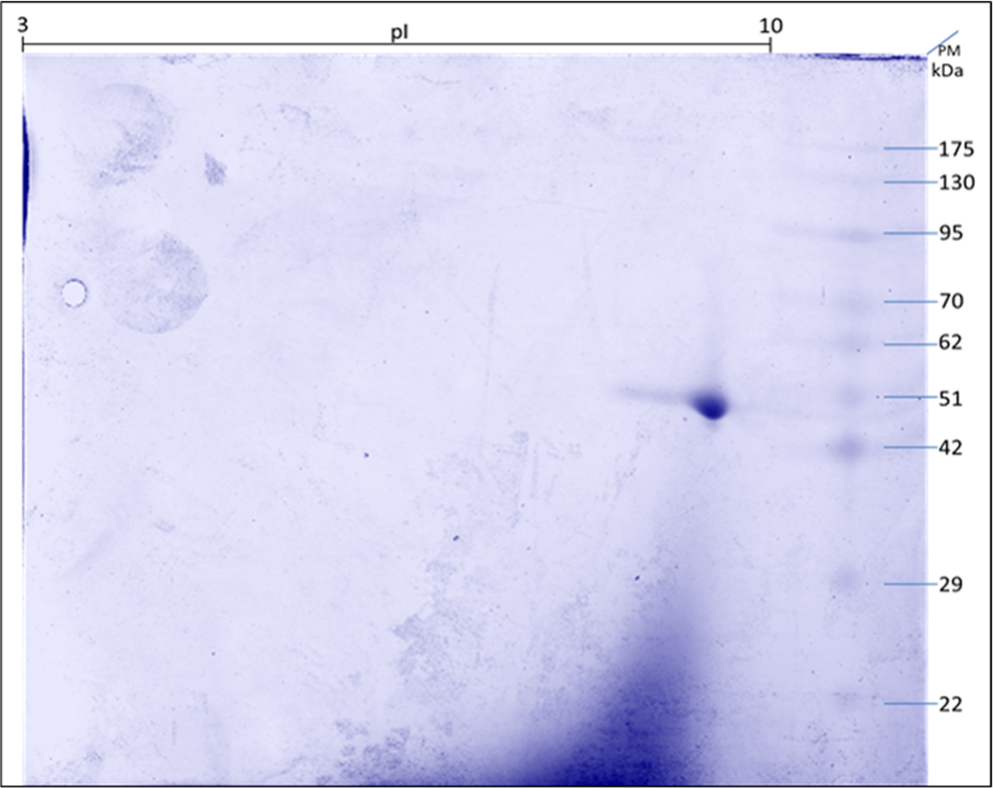
Two-dimensional poly(acrylamide gel electrophoresis) (2D SDS-PAGE, 12%) of the purified PfENR obtained from reversed-phase C18 chromatography.

The PfENR protein (peak 1) was quantified using a highly sensitive DC protein kit, revealing a remarkable yield of approximately 28 mg/L following expression. To evaluate the purity and determine the isoelectric point of PfENR, the collected peak 1 fraction obtained from the C18 reversed-phase chromatography was subjected to a comprehensive two-dimensional poly(acrylamide gel) analysis under reducing conditions (2D 12% SDS-PAGE), as shown in Figure 11. The observed molecular weight of the spot displayed on the 2D SDS-PAGE gel corresponded to approximately 50 kDa, confirming its identity as PfENR. The isoelectric point (pI) of PfENR was determined to be 9.11 through a theoretical calculation using the ProtParam tool available on the ExPASy Bioinformatics Resource Portal, which was in concordance with the experimental pI value obtained through 2D SDS-PAGE analysis.

### 3.6 PfENR Inhibition via Enzymatic Assays

**Figure 12:**
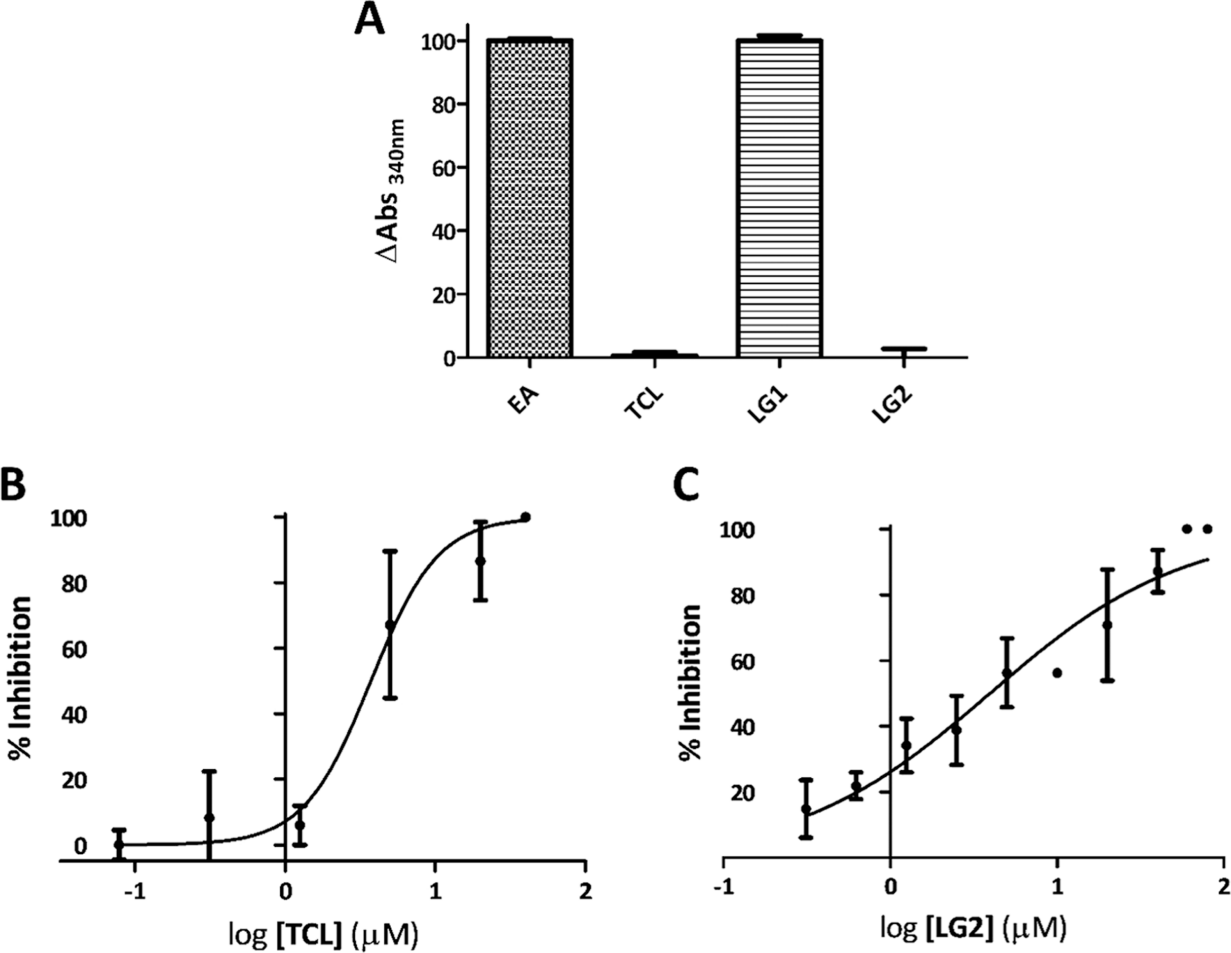
(A) Enzymatic activity of PfENR (EA) and inhibitory activity of ligands TCL, LG1, and LG2 at a concentration of 50 μM. (B) IC50 curves for PfENR inhibition by TCL and (C) LG2. The IC50 values for TCL and LG2 are 3.58 and 3.95 μM, respectively.

In vitro assays were conducted to assess the inhibitory potential of LG1 and LG2 ligands on the PfENR activity. The activity of the PfENR enzyme was measured by monitoring the NADH consumption reaction at 340 nm, and the resulting change in absorbance was normalized to 100% (Figure 12A). Triclosan (TCL) was included as a positive control to establish a baseline, and it showed no significant alteration in NADH consumption, indicating inhibition of PfENR (Figure 12A). LG1 and LG2 were evaluated at a final concentration of 50 μM. The results revealed that LG1 exhibited no detectable inhibitory activity against PfENR, while LG2, at a concentration of 50 μM, completely inhibited PfENR activity.

Further inhibitory activity assays of LG1 and LG2 ligands and the standard TCL revealed that only LG2 exhibited activity against PfENR at a concentration of 50 μM. The IC50 concentrations were determined by using a TCL (Figure 12B) and LG2 (Figure 12C) inhibition assay at varying concentrations. The IC50 values for TCL and LG2 were 3.58 and 3.95 μM, respectively.

Triclosan (TCL) is known to inhibit the *E. coli* ENR^72^ and PfENR enzymes by binding to their acyl substrate-binding pocket.^73^ Previous studies have shown that TCL derivatives also exhibit inhibition of PfENR, with interactions similar to those observed in our study.^56,57^

In terms of the methodology, the combination of computational and experimental approaches proved effective in this study. The molecular docking simulations provided valuable insights into the binding affinities and interactions of the ligands with PfENR, guiding the selection of lead compounds for further evaluation. The subsequent enzymatic assays confirmed the inhibitory potential of LG2 and highlighted its potency in inhibiting PfENR activity. This integrated approach demonstrates the significance of combining computational and experimental techniques in the drug discovery process as it allows for a more comprehensive understanding of ligand–protein interactions and enables the identification of promising drug candidates.

## 4 Conclusions

In conclusion, this investigation elucidates the effectiveness of computational methods in identifying potential drug candidates that selectively target the enoyl-ACP reductase (ENR) of *P. falciparum*. Furthermore, a molecular dynamics simulation with an appropriate time scale is an essential step for validating the docking results. Docking alone cannot conclusively determine which ligands remain stably bound to the receptor. We emphasize the importance of in silico assays accompanied by in vitro molecular confirmation with the specific receptor. This work conducted in vitro molecular assays with stringent control. Based on this study, LG2 emerges as a promising drug candidate, demonstrating in vitro inhibition results akin to those exhibited by triclosan, thereby offering considerable potential as a novel therapeutic option for combating malaria. These compelling results pave the way for further comprehensive investigations encompassing in vitro and in vivo cell testing utilizing more intricate models. The ramifications of this work are far-reaching, as it contributes significantly to the global fight against malaria and the enhancement of public health, offering a promising trajectory toward mitigating the impact of this relentless and devastating disease. Furthermore, a molecular dynamics simulation with an appropriate time scale is an essential step for validating docking results. Docking alone cannot conclusively determine which ligands remain stably bound to the receptor. We emphasize the importance of in silico assays being accompanied by in vitro molecular confirmation with the specific receptor. This work conducted in vitro molecular assays with stringent control.

## Data Availability

The data underlying this study are openly available in Zenodo at 10.5281/zenodo.8302852.

## Supporting Information Available

Triclosan-derived molecules that have been tested in vitro in studies in the literature that have related IC50 values and binding energy made in Autodock vina (Table S1); templates from the Blast search results with the best PfENR structure scores (Table S2); ligands from Library 1 that exhibited the best results in molecular docking to select lead molecules (Table S3); intermolecular interactions between the ligands and 2-*trans*-enoyl-ACP reductase amino acids of *P. falciparum* (PfENR) in the molecular docking performed by the Autodock Vina program (Table S4); analysis of the PfENR structure (Figure S1); validation of the Ramachandran plot statistics for the modeled protein structure using PROCHECK (Figure S2); validation of the predicted in silico model of PfENR by ProSA and QMEAN (Figure S3); RMSD values as a function of time [ns] (Figure S4); graph of the average RMSF values in nanometers for each residue of the structures (Figure S5); and superimposed structures show a visual comparison between the modeled structure and the original 4IGE and 3AM5 structures (Figure S6) (PDF)

## Abbreviations

AUC: area under the curve
BBLAST: basic local alignment search tool
CHJ: ligand 7-(4-chloro-2-hydroxyphenoxy)-4-methyl-2*H*-chromen-2-one
DOPE: discrete optimized protein energy
EA: enzyme activity
ENR: enoyl-ACP reductase
FAS: fatty acid synthesis
GAFF: generalized amber force field
GMOs: genetically modified organisms
H-bond: hydrogen bonds
HIS: histidine tag
IDR: intrinsically disordered region
IPTG: isopropyl-d-1-thiogalactopyranoside
ITNs: methodologies involving a new generation of insecticides
LB: Luria–Bertani
LBDD: ligand-based drug design
LD1: lead compound 1
LD2: lead compound 2
LG1: ligand 1
LG2: ligand 2
MD: molecular dynamics
MM/GBSA: molecular mechanics generalized born surface area
NCBI: national center for biotechnology information
NMR: nuclear magnetic resonance
PDB: Protein Data Bank
PfENR: *P. falciparum* 2-trans-enoyl-ACP reductase
PME: particle mesh ewald
RMSD: root-mean-square deviation
RMSF: root-Mean-square fluctuation
ROC: receptor operational characteristic curve
SBDD: structure-based drug design
SDS-PAGE 12%: sodium dodecyl sulfate-polyacrylamide gel electrophoresis 12%
TCL: triclosan
TN: true negatives
TP: true positives
VS: virtual screening
WHO: World Health Organization
