# Supplementary material for: Exploring Novel Antimalarial Compounds Targeting Plasmodium falciparum Enoyl-ACP Reductase: Computational and Experimental Insights
Source: ACS Omega. 2024 May 13;9(21):22777–93. doi: 10.1021/acsomega.3c09893 (PMC11137734; doi:10.1021/acsomega.3c09893)
Supplement: Supplementary file 1 — ao3c09893_si_001.pdf [file ao3c09893_si_001.pdf]

Supporting Information

Exploring Novel Antimalarial Compounds Targeting *Plasmodium falciparum* Enoyl-ACP Reductase: Computational and Experimental Insights

George A. R. Oliveira[a,e], Bruno G. D. V. Morales[a,b], Rosa M. O. Sousa[d], Soraya S. Pereira[b,d,e], Deborah Antunes[f], Ernesto R. Caffarena[e,g] e Fernando B. Zanchi[a,b,c,e]*

[a]Laboratório de Bioinformática e Química Medicinal, Fundação Oswaldo Cruz, CEP: 76812-245, Porto Velho-RO, Brazil;

[b]Programa de Pós-Graduação em Biologia Experimental, Fundação Universidade Federal de Rondônia (UNIR), CEP: 76801-974, Porto Velho-RO;

[c]Instituto Nacional de Epidemiologia na Amazônia Ocidental - EPIAMO, CEP: 76812-245, Porto Velho-RO, Brazil;

[d]Laboratório de Engenharia de Anticorpos, Fundação Oswaldo Cruz de Rondônia, CEP: 76812-245, Porto Velho-RO, Brazil;

[e]Programa de Pós-graduação Stricto sensu em Biologia Computacional e Sistemas do Instituto Oswaldo Cruz, CEP: 21040-360, Rio de Janeiro-RJ, Brazil (PGBCS/IOC);

[f]Laboratório de Genômica Aplicada e Bioinovações, Instituto Oswaldo Cruz, Fundação Oswaldo Cruz (FIOCRUZ), CEP: 21040-900, Rio de Janeiro-RJ, Brazil;

[g]Programa de Computação Científica - PROCC. Fundação Oswaldo Cruz, CEP: 21040-900, Rio de Janeiro-RJ. Brazil.

*Email: george.oliveira92@gmail.com

SUPPLEMENTARY MATERIAL

Table S1: triclosan-derived molecules that have been tested in vitro in studies in the literature that have related IC50 values and binding energy made in Autodock vina.

Order	CID	IC50 (mM)	Binding energy (kcal.mol-1)	Activity
1	25023968	0.038	-10.1	Yes[3]
2	25023955	0.049	-9.6	Yes[3]
3	5564	0.050	-9.2	Yes[4]
4	11674015	0.057	-9.1	Yes[1]
5	25023969	0.071	-9.4	Yes[3]
6	16220128	0.076	-10.2	Yes[3]
7	25023958	0.110	-9.3	Yes[3]
8	11659169	0.120	-9.6	Yes[1]
9	44405331	0.120	-9.4	Yes[1]
10	25023963	0.120	-8.5	Yes[3]
11	11660481	0.140	-9.8	Yes[1]
12	44405338	0.140	-9.5	Yes[1]
13	25023954	0.140	-9.8	Yes[3]
14	44405339	0.160	-9.7	Yes[1]
15	44405293	0.170	-9.8	Yes[1]
16	6852143	0.180	-9.3	Yes[1]
17	25023961	0.180	-8.7	Yes[3]
18	44405311	0.190	-9.5	Yes[1]
19	25023967	0.190	-10.3	Yes[3]
20	44405291	0.200	-10.3	Yes[1]
21	22947105	0.200	-9.6	Yes[3]
22	25023959	0.210	-8.4	Yes[3]
23	21272512	0.220	-8.8	Yes[1]
24	25023966	0.230	-8.9	Yes[3]
25	71768351	0.250	-10.0	Yes[4]
26	6914566	0.250	-9.8	Yes[1]
27	73347724	0.270	-9.3	Yes[4]
28	25023962	0.290	-8.4	Yes[3]
29	6914565	0.310	-9.6	Yes[1]
30	44405380	0.320	-10.2	Yes[1]
31	6852148	0.360	-8.8	Yes[1]

**32**	44405287	0.370	-10.0	Yes[1]
**33**	25023965	0.410	-9.3	Yes[3]
**34**	44405330	0.413	-9.6	Yes[1]
**35**	15942656	0.440	-9.4	Yes[3]
**36**	16220126	0.440	-10.2	Yes[3]
**37**	72703246	0.450	-10.9	Yes[4]
**38**	44405274	0.460	-9.9	Yes[1]
**39**	25023960	0.480	-8.2	Yes[3]
**40**	25023964	0.530	-9.1	Yes[3]
**41**	25023973	0.530	-9.7	Yes[3]
**42**	44405327	0.550	-9.2	Yes[1]
**43**	44405314	0.560	-9.8	Yes[1]
**44**	44405289	0.590	-10.6	Yes[1]
**45**	72703044	0.630	-9.8	Yes[4]
**46**	16220130	0.640	-9.6	Yes[3]
**47**	44405276	0.680	-10.3	Yes[1]
**48**	16220129	0.770	-9.5	Yes[3]
**49**	44405275	0.810	-10.5	Yes[1]
**50**	25023972	0.840	-9.2	Yes[3]
**51**	44405298	1.200	-10.0	Yes[1]
**52**	44405336	2.500	-11.0	Yes[1]
**53**	44410287	2.500	-8.8	Yes[2]
**54**	73355324	2.700	-10.3	Unspecified[4]
**55**	25023971	2.800	-7.8	Unspecified[3]
**56**	44405271	3.000	-10.3	Unspecified[1]
**57**	44410130	3.300	-8.2	Unspecified[2]
**58**	72703050	5.500	-8.8	Unspecified[4]
**59**	71768352	6.000	-10.5	Unspecified[4]
**60**	44410234	6.100	-8.2	Unspecified[2]
**61**	44410251	7.000	-8.6	Unspecified[2]
**62**	44410094	7.200	-8.1	Unspecified[2]
**63**	44410256	7.200	-7.1	Unspecified[2]
**64**	11495355	9.000	-8.5	Unspecified[2]
**65**	44410284	10.000	-8.8	Unspecified[2]
**66**	72703046	11.000	-9.6	Unspecified[4]
**67**	44410086	11.000	-8.6	Unspecified[2]
**68**	44410238	11.000	-9.8	Unspecified[2]
**69**	72703244	12.000	-9.4	Unspecified[4]

**70**	72703242	14.000	-10.2	Unspecified[4]
**71**	72703045	14.200	-9.8	Unspecified[4]
**72**	72703245	15.000	-9.8	Unspecified[4]
**73**	44410170	15.000	-8.3	Unspecified[2]
**74**	44410133	18.000	-8.6	Unspecified[2]
**75**	44410081	19.000	-8.9	Unspecified[2]
**76**	44410134	19.000	-7.0	Unspecified[2]
**77**	25023957	21.000	-8.5	Unspecified[3]
**78**	44410066	22.000	-8.3	Unspecified[2]
**79**	44410291	26.000	-8.8	Unspecified[2]
**80**	11948630	27.000	-7.9	Unspecified[2]
**81**	44410098	27.000	-8.4	Unspecified[2]
**82**	44410097	28.000	-7.9	Unspecified[2]
**83**	25023970	33.000	-9.6	Unspecified[3]
**84**	44410210	35.000	-10.2	Unspecified[2]
**85**	44410286	35.000	-9.0	Unspecified[2]
**86**	72703243	44.000	-8.4	Unspecified[4]
**87**	44410128	46.000	-9.6	Unspecified[2]
**88**	44410129	50.000	-8.9	Unspecified[2]
**89**	44410138	50.000	-7.5	Unspecified[2]
**90**	44410211	50.000	-8.9	Unspecified[2]
**91**	44410215	50.000	-8.4	Unspecified[2]
**92**	44410233	50.000	-7.8	Unspecified[2]
**93**	44410252	50.000	-8.4	Unspecified[2]
**94**	44410295	50.000	-8.0	Unspecified[2]
**95**	23656593	100.000	-8.4	Unspecified[3]
**96**	25023956	100.000	-9.4	Unspecified[3]

[1] (Freundlich, 2005); [2] (Freundlich, 2006); [3] (Freundlich, 2007); [4] (Belluti, 2013).

Table S2: Templates from the Blast search results with the best PfENR structure scores.

Species	PDB (id)	Resolution (Å)	Identity (%)	Query cover (%)	Ligand
P. falciparum 3D7	4IGE	2.15	100%	78%	*7-(4-chloro-2-hydroxyphenoxy) -4-methyl-2h-chromen-2-one* (CHJ)
P. falciparum	1VRW	2.40	100%	77%	-----
P. falciparum	2OL4	2.26	100%	76%	*2-(2,4-dichlorophenoxy) -5-(3-phenylpropyl) phenol*
P. falciparum 3D7	2FOI	2.50	100%	62%	*4-(2,4-dichlorophenoxy) -2'-methylbiphenyl-3-ol*
P. falciparum	1NHG	2.43	100%	53%	Triclosan
P. falciparum	3AM5	2.05	99.9%	76%	Triclosan
P. falciparum	3AM3	2.50	99.9%	76%	Triclosan
P. falciparum	2O2Y	2.20	99.7%	80%	Triclosan
P. falciparum	1UH5	2.20	99.4%	76%	Triclosan

Table S3: Ligands from Library 1 that exhibited the best results in molecular docking to select lead molecules.

Item	CID	2D Structure	Molecular Formula	Binding Energy (kcal/mol)
1	44405336	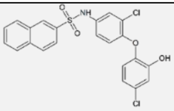	$C_{22}H$ $_{5}Cl_2NO_4S$	-11.0
2	72703246	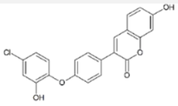	$C_{21}H_{13}ClO_5$	-10.9
3	44405289	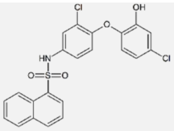	$C_{22}H_{15}Cl_2NO_4S$	-10.6
4	71768352	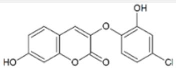	$C_{15}H_9ClO_5$	-10.5
5	44405275	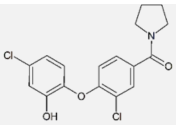	$C_{17}H_{15}Cl_2NO_3$	-10.4
6	25023967	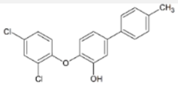	$C_{19}H_{14}Cl_2O_2$	-10.3
7	44405271	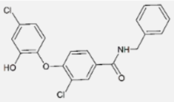	$C_{20}H_{15}Cl_2NO_3$	-10.3
8	71768351 (CHJ)	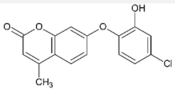	$C_{16}H_{11}ClO_4$	-10.0
9	5564 (TCL)	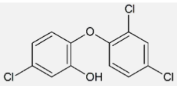	$C_{12}H_7Cl$ O_2	-9.2

Table S4: Intermolecular interactions between the ligands and 2-*trans*-enoyl-ACP reductase amino acids of *P. falciparum* (*Pf*ENR) in the molecular docking performed by the Autodock Vina program.

Ligand (CID)	*Pf*ENR or Cofactor	Types	Category	Binding energy (kcal/mol)
**CHJ (71768351)**	Try277, NAD^+	Conventional Hydrogen Bond	Hydrogen Bond	-10.0
	NAD^+	Pi-Cation	Electrostatic	
	Ala217	Pi-Sigma	Hydrophobic	
	Tyr267	Pi-Pi T-shaped	Hydrophobic	
	Ala219, Val222, Met281, Ile369	Alkyl	Hydrophobic	
	Ala217, Ala319, Met281, Ala320, NAD^+, Tyr267, Phe368	Pi-Alkyl	Hydrophobic	
**TCL (5564)**	Tyr277, NAD^+	Conventional Hydrogen Bond	Hydrogen Bond	-9.2
	NAD^+	Pi-Cation	Electrostatic	
	Tyr267	Pi-Pi T-shaped	Hydrophobic	
	Pro314, Ile369, Ala217, Ala319, Ala219, Val222, Met281	Alkyl	Hydrophobic	
	Ala320, NAD^+, Ala217, Met281, Ala319, Ile323, Tyr267, Phe368	Pi-Alkyl	Hydrophobic	
**LD1 (44405336)**	Asp218	Conventional Hydrogen Bond	Hydrogen Bond	-11.0
	NAD^+	Carbon Hydrogen Bond	Hydrogen Bond	
	Val222	Pi-Sigma	Hydrophobic	
	Trp131	Pi-Sulfur	Other	

	Ala319	Alkyl	Hydrophobic	
	Arg318, Ala319, Ala322	Pi-Alkyl	Hydrophobic	
**LD2 (72703246)**	Ala219, NAD^+	Conventional Hydrogen Bond	Hydrogen Bond	-10.9
	NAD^+	Pi-Cation	Electrostatic	
	Val222, Ile323	Pi-Sigma	Hydrophobic	
	Tyr267	Pi-Pi T-shaped	Hydrophobic	
	Pro314, Ile369, Ala372, Met281,	Alkyl	Hydrophobic	
	Ala319, Ala219, Ala322, Ala320, NAD^+, Tyr267, Phe368	Pi-Alkyl	Hydrophobic	
**LG1 (4245155)**	Asn218	Conventional Hydrogen Bond	Hydrogen Bond	-11.5
	NAD^+	Carbon Hydrogen Bond	Hydrogen Bond	
	NAD^+	Pi-Cation	Electrostatic	
	NAD^+	Pi-Anion	Electrostatic	
	Ala319	Pi-Sigma	Hydrophobic	
	Tyr267	Pi-Pi T-shaped	Hydrophobic	
	Ala217, Ala319	Alkyl	Hydrophobic	
	Ala217, Val222, Met281, Ala319, Ala322, Arg318, Ala319, Ala320, NAD^+	Pi-Alkyl	Hydrophobic	
**LG2***	NAD^+	Pi-Cation	Electrostatic	-10.8
	Tyr267, Ala217, Ala319,	Pi-Sigma	Hydrophobic	
	Tyr267	Pi-Pi T-shaped	Hydrophobic	
	Pro314, NAD^+, Met281,	Alkyl	Hydrophobic	

	Ala319, Ala322, Ala320, NAD^+	Pi-Alkyl	Hydrophobic

*Ligand that does not have a structure deposited in the PubChem database. It was commercially acquired.

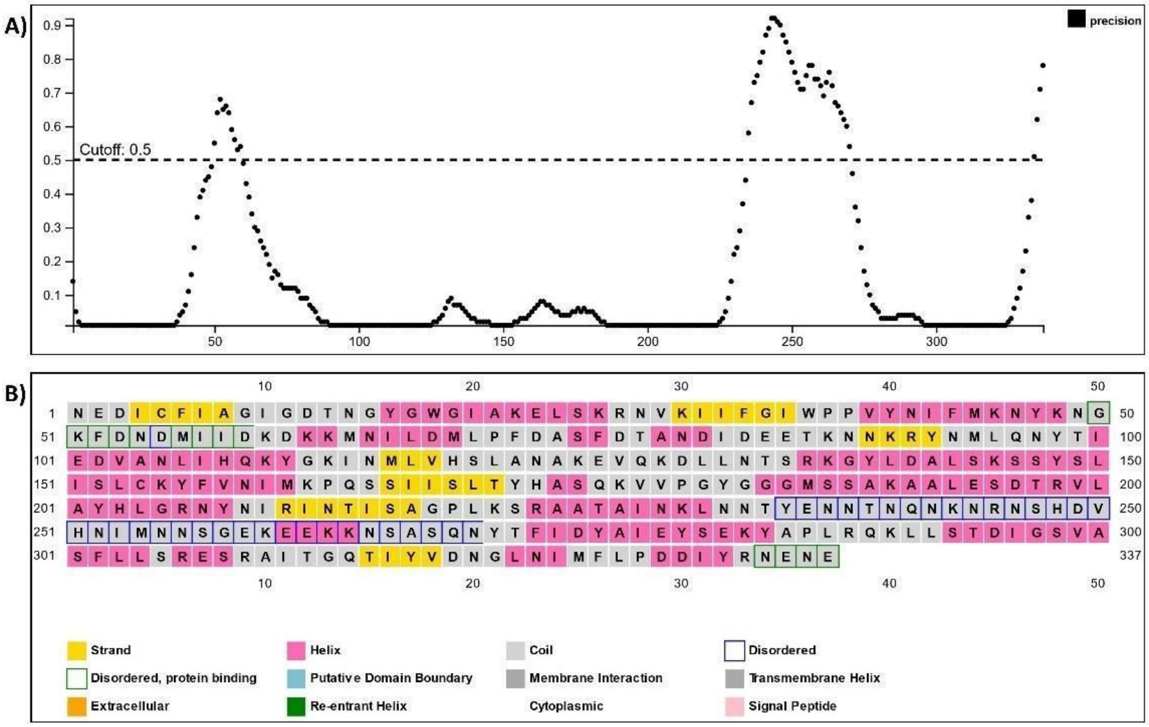

Figure S1: Analysis of the *Pf*ENR structure. (A) Using DISOPRED to assess intrinsically disordered regions (IDRs), and (B) using PSIPRED to evaluate the probability of secondary structures within the IDRs regions.

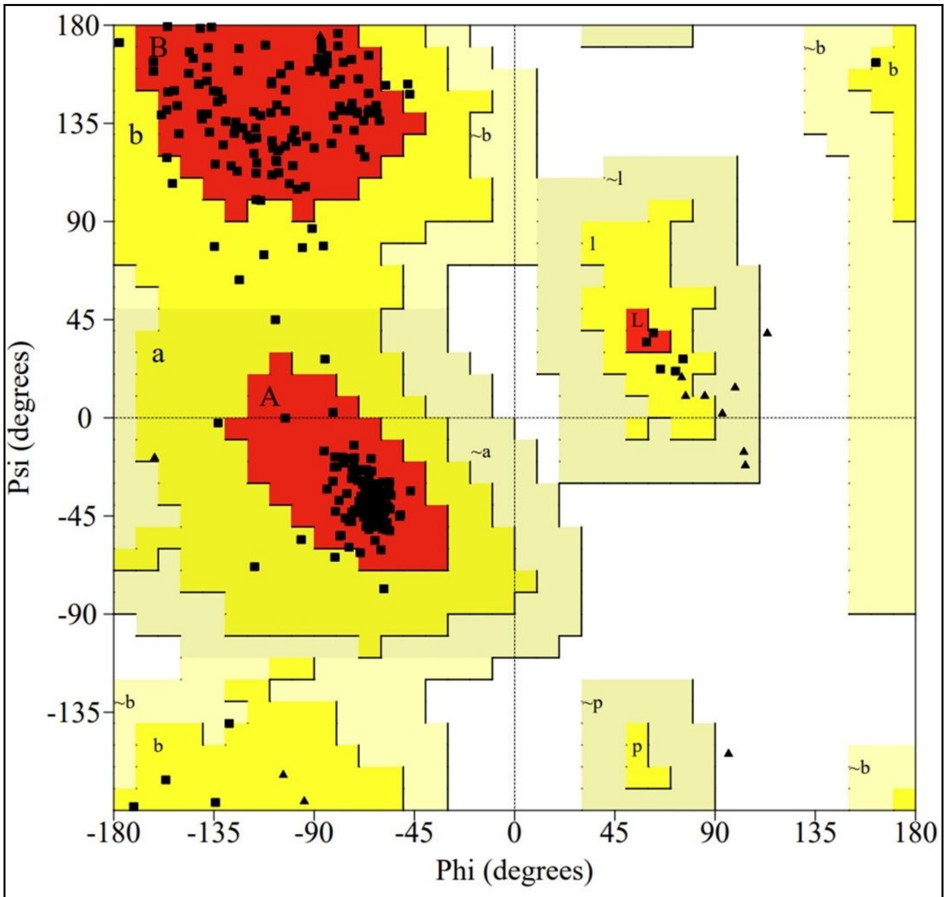

Plot statistics

Residues in most favoured regions [A,B,L]	270	90.3%
Residues in additional allowed regions [a,b,l,p]	29	9.7%
Residues in generously allowed regions [~a,~b,~l,~p]	0	0.0%
Residues in disallowed regions	0	0.0%
	----	------
Number of non-glycine and non-proline residues	299	100.0%
Number of end-residues (excl. Gly and Pro)	2	
Number of glycine residues (shown as triangles)	19	
Number of proline residues	8	

Total number of residues	328	

Figure S2: Validation of Ramachandran Plot Statistics for the Modeled Protein Structure Using PROCHECK. The Ramachandran plot displays the most favored locations in red, while the other allowed areas are in yellow.

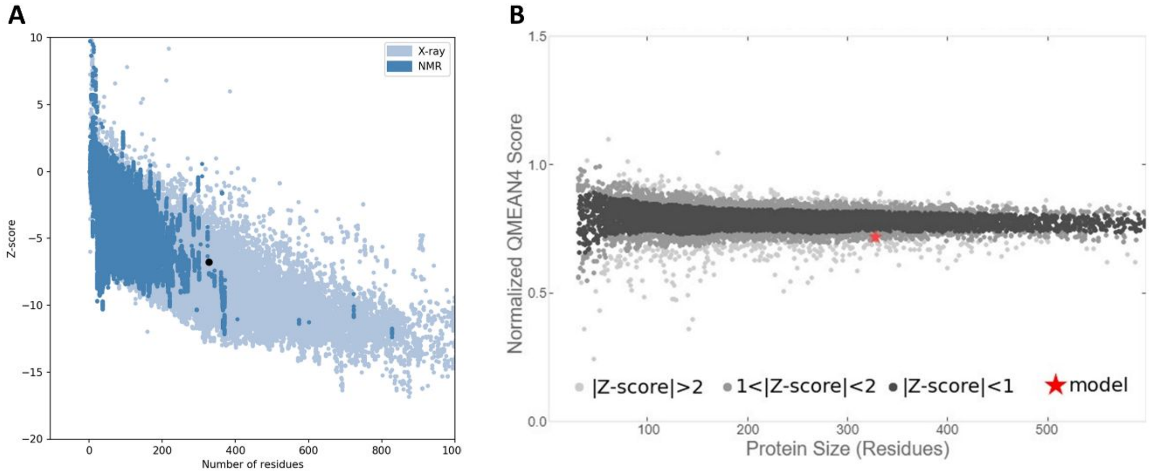

Figure S3: Validation of the predicted in silico model of PfENR by ProSA and QMEAN. (A) ProSA-based Structure Validation of Predicted PfENR Model: Z-score Comparison with Non-redundant Crystallographic Structures (light blue dots) and NMR Structures (dark blue dots) and (B) QMEAN-based Structure Validation of Predicted PfENR Model: QMEAN-score Comparison with Non-redundant High-Resolution Experimental Structures (gray and black dots).

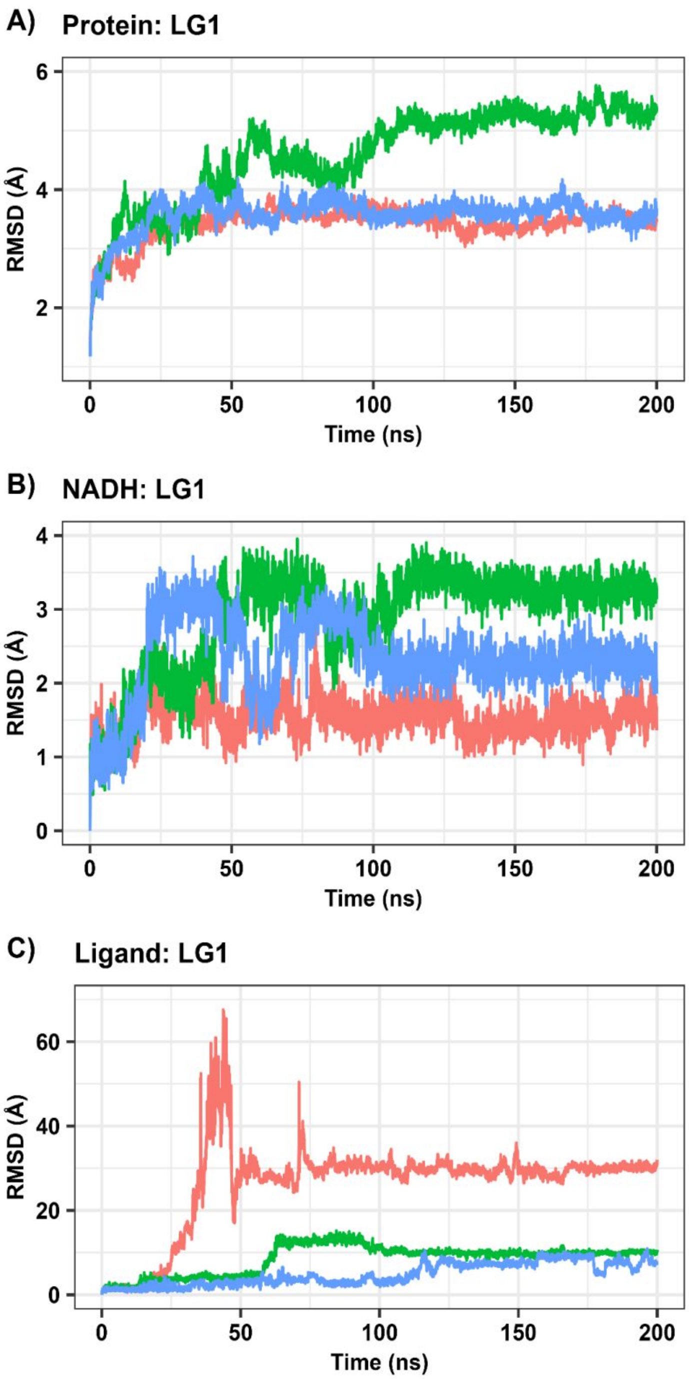

Figure S4: RMSD values as a function of time [ns]. (A) *Pf*ENR from the *Pf*ENR-NADH-LG1 complex; (B) NADH from the *Pf*ENR-NADH-LG1 complex; and (C) LG2 from the *Pf*ENR-NADH-LG1 complex.

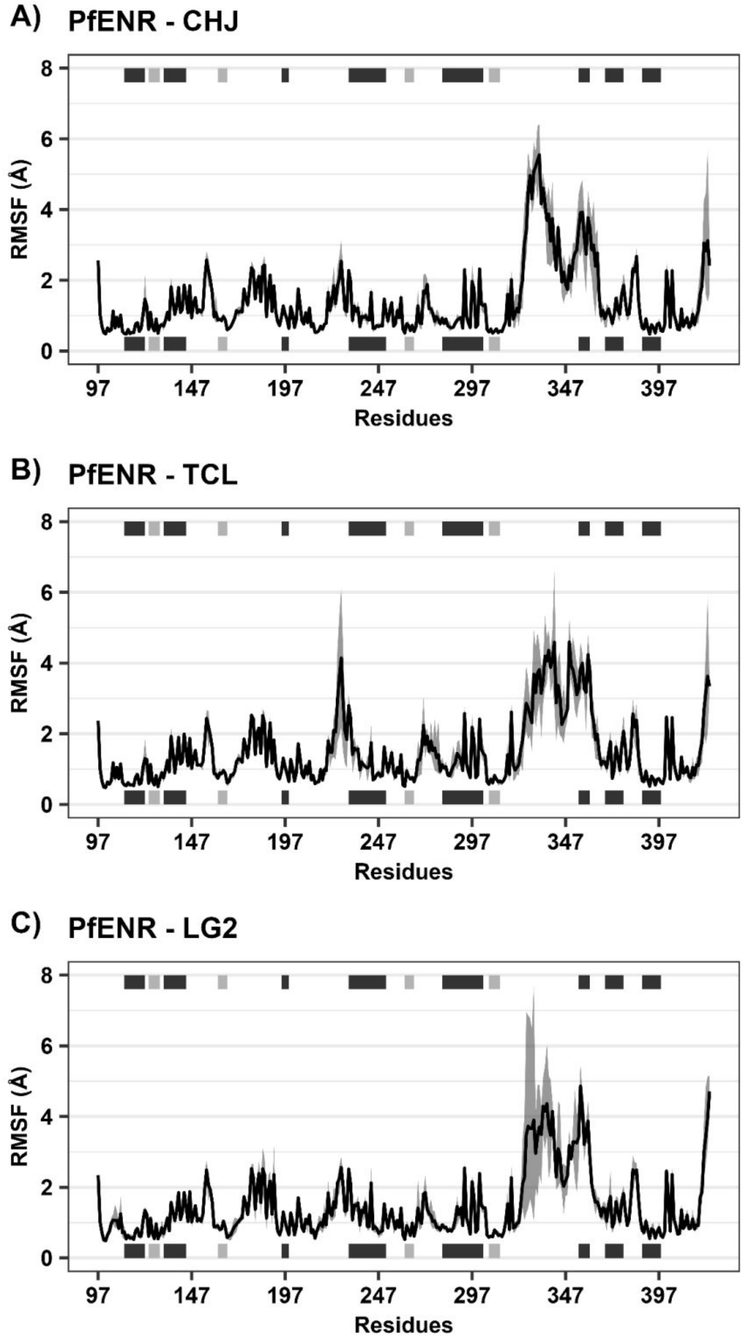

Figure S5: Graph of the average RMSF values in nanometers for each residue of the structures. (A) Average of triplicates for *Pf*ENR-NAD+-CHJ; (B) Average of triplicates for *Pf*ENR-NAD+-TCL; and (C) Average of triplicates for *Pf*ENR-NAD+-LG2.

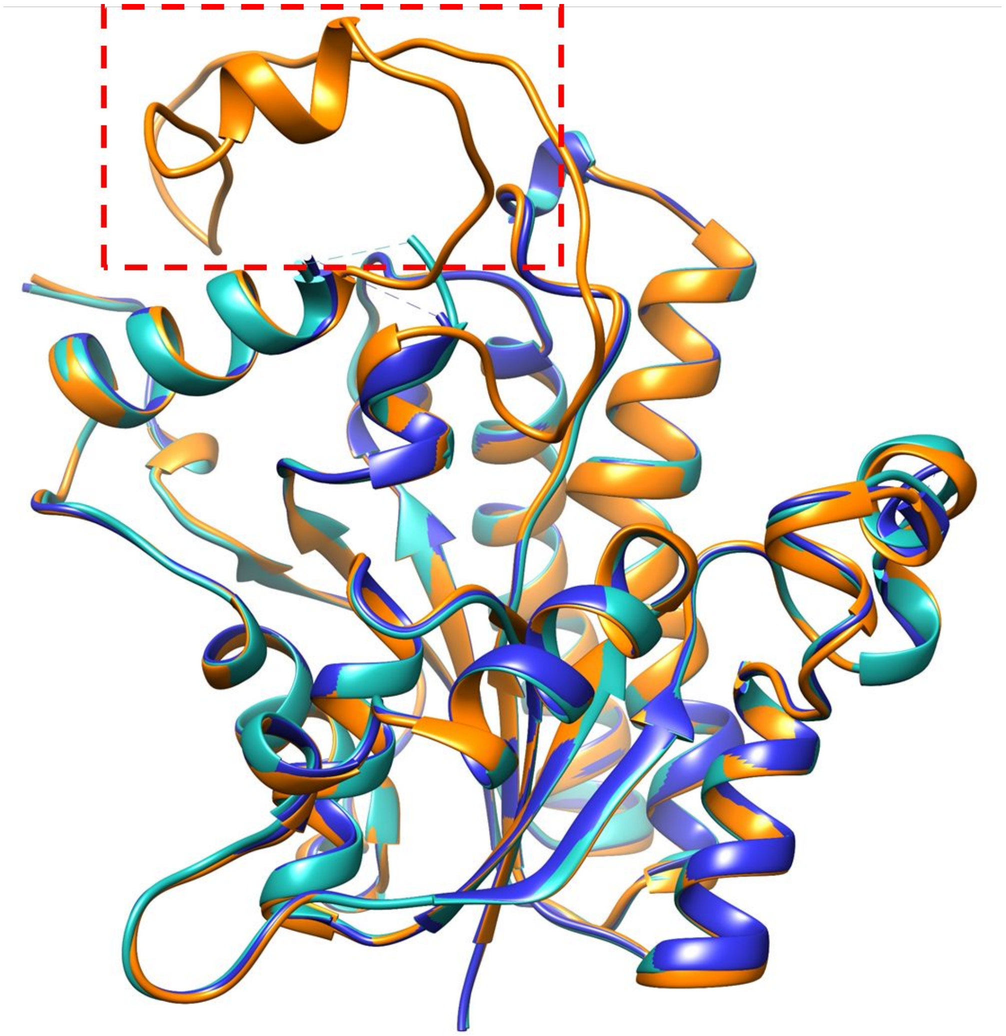

Figure S6: The superimposed structures show a visual comparison between the modeled structure and the original 4IGE and 3AM5 structures. The modeled region is highlighted in red. In this composite representation, the modeled portion is represented in orange, the 3AM5 structure is indicated in green and the 4IGE structure is represented in purple.
